# Synthesis, Characterization, and Density Functional Theory Investigation of the Solid-State [UO_2_Cl_4_(H_2_O)]^2–^ Complex

**DOI:** 10.1021/acs.inorgchem.3c01725

**Published:** 2023-08-23

**Authors:** Harindu Rajapaksha, Sara E. Mason, Tori Z. Forbes

**Affiliations:** † Department of Chemistry, University of Iowa, Iowa City, Iowa 52242, United States; ‡ Center for Functional Nanomaterials, Brookhaven National Laboratory, Upton, New York 11973, United States

## Abstract

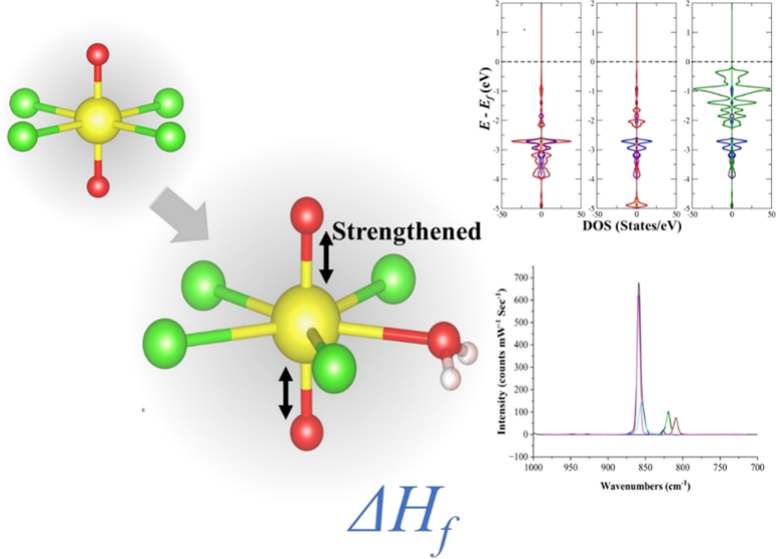

A significant number of solid-state [UO_2_Cl_4_]^2–^ coordination compounds have been synthesized and structurally characterized. Yet, despite their purposive relative abundance in aqueous solutions, characterization of aquachlorouranium(VI) complexes remain rare. In the current study, a solid-state uranyl aqua chloro complex ((C_4_H_12_N_2_)_2_[UO_2_Cl_4_(H_2_O)]Cl_2_) was synthesized using piperazinium as a charge-balancing ligand, and the structure was determined using single-crystal X-ray diffraction. Using periodic density functional theory, the electronic structure of the [UO_2_Cl_4_(H_2_O)]^2–^ complex was compared to [UO_2_Cl_4_]^2–^ to uncover the strengthening of the U=O bond in [UO_2_Cl_4_(H_2_O)]^2–^. Changes in the strength of the U=O bond were validated further with Raman and IR spectroscopy, where uranyl symmetrical (ν_1_) and asymmetrical (ν_3_) stretches were blue-shifted compared to the reference [UO_2_Cl_4_]^2–^ complex. Furthermore, the formation energy of the solid-state (C_4_H_12_N_2_)_2_[UO_2_Cl_4_(H_2_O)]Cl_2_ complex was calculated to be −287.60 ± 1.75 kJ mol^–1^ using isothermal acid calorimetry. The demonstrated higher stability relative to the related [UO_2_Cl_4_]^2–^ complex was related to the relative stoichiometry of the counterions.

## Abstract 2

The electronic structure, bonding, and vibrational spectra of [UO_2_Cl_4_(H_2_O)]^2−^ containing compounds were connected with [UO_2_Cl_4_]^2−^ containing compound. All of our finding suggests stronger U=O in [UO_2_Cl_4_(H_2_O)]^2−^ than [UO_2_Cl_4_]^2−^. The stability of the crystalline phase of [UO_2_Cl_4_(H_2_O)]^2−^ was analyzed using formation enthalpies (Δ*H*_f_)

## 1 Introduction

Solid-state uranyl (U(VI)O_2_^2+^) chloride compounds have been widely used to evaluate fundamental *f*-block chemistry and intermolecular interactions within actinide coordination compounds.^1−6^ Most uranyl chloride solid phases are synthesized through evaporation of U(VI) in an acidic chloride media, which typically leads to the formation of the [UO_2_Cl_4_]^2–^ coordination compounds.^1,4^ Currently, there are 112 structures containing the [UO_2_Cl_4_]^2–^ anions in the CCDC database.^7^ The vast structural catalog of this system provides a basis to explore rational design principles in hybrid actinide compounds.^1,4^ For example, the commonality of the [UO_2_Cl_4_]^2–^ anion enabled a systematic evaluation of noncovalent interactions in solid-state materials that led to insights regarding the crystallization of U(VI) coordination compounds.^1−4,8,9^ In addition, the [UO_2_Cl_4_]^2–^ complex has been crystallized with a range of alkali and organic cations and used to determine the optical,^1^ vibrational,^3,5,6^ and thermodynamic^2,9,10^ properties of these phases to provide insights into the nature of bonding within the 5*f* system.

While the [UO_2_Cl_4_]^2–^ complex is prevalent in the solid state, there is a range of possible complexes in solution that exist with the general formula of [UO_2_Cl*_n_*(H_2_O)_5–*n*_]^2–*n*^.^11,12^ The pentaaqua uranyl complex, [UO_2_(H_2_O)_5_]^2+^, is the most prevalent species when [Cl^–^] < 2 M.^13−15^ As [Cl^–^] increases beyond 2 M, the [UO_2_(H_2_O)_5_]^2+^ undergoes a ligand interchange reaction to form [UO_2_Cl (H_2_O)_4_]^+^.^12,16−18^ Increasing [Cl^–^] to 4–6, 6–10, and >14 M results in the formation of [UO_2_Cl_2_(H_2_O)_3_]^0^, [UO_2_Cl_3_(H_2_O)_2_]^−^, and [UO_2_Cl_4_]^2–^, respectively.^12,15,18^ Stability of these mixed aquachlorouranium(VI) complexes has been previously studied by Bühl et al. using Density Function Theory (DFT), and they reported energetic favorability for chloride complexation when U(VI) is modeled with polarizable continuum solvation.^19^ However, Bühl et al. also noted that modeling the speciation of aquachlorouranium(VI) complexes is difficult because of small free-energy differences between the various species. They determined that coordination complexes with higher chloride coordination such as [UO_2_Cl_4_]^2–^ are metastable in pure water.^19^ Soderholm and colleagues explored the U(VI) chloride system using pair distribution function analysis and determined that the number of ligands directly coordinating with uranyl decreases as [Cl^–^] increases from 2 to 7 M. Overall, they noted that the total average coordination in the uranyl equatorial plane is about 4.4 as [Cl^–^] approaches 7 M, suggesting a mix of tetra- and pentachloro species in solution. Increasing [Cl^–^] increased the number of bound Cl^–^, but even at high chloride concentrations, an average of 1.7 ligated water molecules were still associated with the U(VI) complex.^12^ Similar observations were noted by Allen and co-workers, indicating that hydrated uranyl chloride species are prevalent in the solution phase.^15^

Compared to the [UO_2_Cl_4_]^2–^ coordination complexes, the existence of aquachlorouranium(VI) complexes in solid-state compounds is much more rare, with only a handful of reported structures containing isolated molecular units. Only one other monomeric species has been structurally characterized: the [UO_2_Cl_2_(H_2_O)]^0^ complexes, identified using powder X-ray diffraction by Debets.^20^ Dimeric species that result from hydrolysis of the U(VI) cation have also been reported,^21^ and Cahill et al. described the synthesis and characterization of a solid-state [UO_2_Cl_3_(H_2_O)(pyrazine)_0.5_]^2–^ complex that contains a bridging pyrazine molecule between the two metal centers.^22^ Larger tetranuclear (VI) complexes containing bridging hydroxide and chloride anions have been reported by Aberg,^23^ but no additional solid phases have been reported for aquachlorouranium(VI) coordination compounds. More specifically, the [UO_2_Cl_4_(H_2_O)]^2–^ complex has not yet been isolated in the solid state and represents a unique opportunity to analyze the effect of water coordination on the axial bond, electronic structure, and vibrational properties of uranyl aqua chloro complexes in contrast to the well-studied [UO_2_Cl_4_]^2–^ anion. Charge-balancing organic cations have been utilized extensively in the isolation of uranyl chloro complexes as a result of their ability to form charge-assisted hydrogen bonds, which aid in crystallization.^1,3,6,9^ Due to its ability to form multiple charge-assisted hydrogen bonds with the uranyl halide moiety, the piperazinium cation (C_4_H_12_N_2_)^2+^ has been demonstrated to be highly effective in crystallizing actinyl halide complexes.^9,24^

Herein, we report the synthesis and structural analysis of the [UO_2_Cl_4_(H_2_O)]^2–^ complex that has been isolated within the (C_4_H_12_N_2_)_2_[UO_2_Cl_4_(H_2_O)]Cl_2(s)_ compound. We used periodic Density Functional Theory (DFT) to probe the primary coordination sphere, electronic structure, and hydrogen bonding of [UO_2_Cl_4_(H_2_O)]^2–^ in contrast with [UO_2_Cl_4_]^2–^. Additionally, Raman and IR spectroscopy were utilized to probe into the uranyl vibrational modes for this complex. Finally, isothermal acid calorimetry was performed to assess the stability of the solid-state (C_4_H_12_N_2_)_2_[UO_2_Cl_4_(H_2_O)]Cl_2_ phase and explore the impacts of the ligated water on the crystallization of uranyl chloride coordination compounds.

## 2 Experimental Methods

### 2.1 Synthesis of (C_4_H_12_N_2_)_2_[UO_2_Cl_4_(H_2_O)]Cl_2(s)_

All reagents were used as received. *Caution: Uranyl acetate dihydrate ((UO*_2_*)(CH*_3_*COO)*_2_·2*H*_*2*_*O) used in this study contains U-238; standard precautions and licensing for handling radioactive substances should be followed.* Crystalline materials were synthesized by adding 120 mg of uranyl acetate dihydrate (International Bio-analytical Industries, 98–102%) into 3.00 mL of methanol in a 20 mL scintillation vial. An additional 2.00 mL of Millipore water and 1.00 mL of concentrated (12 N) HCl were added to the vial, followed by the addition of 3.00 mL of solution containing 0.4 M piperazine in H_2_O. The reaction solution was mixed with a magnetic stir bar before undergoing an evaporation step at 120 °C until the solution volume was reduced to 1 mL. Clear yellow crystals formed at the bottom of the vial within 48 h of slow evaporation (opening covered with a perforated parafilm) with a yield of 80–90% based upon U. Additional details, including the synthesis optimization is provided in the Supporting Information (Table S1).

### 2.2 Single-Crystal X-ray Diffraction

Individual crystals were extracted from the mother liquor, and a high-quality single crystal of the material was placed on a Mitigen micromount. Reflections were acquired using 0.5° ω scans at 100 K on a Bruker D8 Quest single X-ray diffractometer (λ_Mo Kα_ = 0.71073 Å) equipped with a PHOTON detector and an Oxford cryo-system operating at 100 K. Data collection and integration of the data was performed using the Bruker APEX3 software and absorption corrections were performed using the SADABS program. The initial structure was solved using APEX3 intrinsic phasing, and least squares refinements of the partial structure model were performed using SHELXL and the OLEX2^25^ software packages. Hydrogen atoms associated with the piperazinium cation and water molecules were modeled with AFIX 23 and AFIX 7, respectively. Select crystallographic information for (C_4_H_12_N_2_)_2_[UO_2_Cl_4_(H_2_O)]Cl_2(s)_ can be found in Table 1, and the thermal ellipsoid plot is available in the Supporting Information (Figure S1). The crystallographic information file (CIF) deposition number is 2241014 and can be obtained free of charge from The Cambridge Crystallographic Data Centre via www.ccdc.cam.ac.uk/data_request/cif.

**Table 1:** Selected Crystallographic Parameters of (C_4_H_12_N_2_)_2_[UO_2_Cl_4_(H_2_O)]Cl_2(s)_

empirical formula	(C_4_H_12_N_2_)_2_[UO_2_Cl_4_(H_2_O)]Cl_2_	μ(mm^–1^)	8.731
crystal color and habit	yellow prismatic	F(000)	1276.0
formula weight	676.04	Θ range (°)	4.504–50.692
crystal system	orthorhombic	limiting indices	–14 ≤ *h* ≤ 14
*a* (Å)	12.2130(7)		–14 ≤ *k* ≤ 14
*b* (Å)	12.4456(7)		–16 ≤ *l* ≤ 16
*c* (Å)	13.4579(8)	ref collected/unique	100 055
α (°)	90°	*R*_int_	0.0621
β (°)	90°	data/restraints/parameters	1953/0/114
γ (°)	90°	GOF on *F*^2^	1.198
volume (Å^3^)	2045.6(2)	final *R* indices ([*I* > 2σ(*I*)]) *R*_1_	0.0113
temperature (K)	102.07	final *R* indices ([*I* > 2σ(*I*)]) *wR*_2_	0.0266
density, ρ (g cm^–3^)	2.195	*R* indices (all data) R_1_	0.0116
space group	**Pnma**	*R* indices (all data) *w*R_2_	0.0267
*Z*	4	largest diff. peak/hole (e Å^–3^)	0.51 and −0.98
radiation type	Mo Kα (λ = 0.71073)	CCDC deposition number	2 241 014

### 2.3 Vibrational Spectroscopy

Solid-state Raman spectroscopy was collected on polycrystalline materials using a SnRI High-Resolution Sierra 2.0 Raman spectrometer outfitted with a 786 nm laser and a 2048 pixel TE-CCD. The laser intensity was set to 15 mW, and spectra were obtained with a 5s integration period. FT-IR spectra of solid-state materials were acquired using a Brucke^r^ Vertex 70v with a HeNe laser of 633 nm energy and 1 mW power. The FT-IR spectra were acquired at a final pressure of 10^–5^ mbar using 64 backgrounds and 64 sample scans ranging from 4000 to 400 nm.

### 2.4 Calorimetry

The purity of the crystalline material was analyzed through powder X-ray diffraction and combustion elemental analysis prior to calorimetric measurements (Supporting information, Section 5: Figure S2 and Table S5). The solvation enthalpy of (C_4_H_12_N_2_)_2_[UO_2_Cl_4_(H_2_O)]Cl_2(s)_ was determined using a Setatram Calvet C80 calorimeter at 25.0 ± 0.1 °C and ambient pressure. Depending on the signal-to-noise ratio, 10–30 mg of pure samples were mixed with 1.000 mL of HCl solution (2N HCl in H_2_O), and the isothermal heat transfer rate was recorded and compared to the reference. To achieve the appropriate equilibration of the system, a baseline variation of <0.05 mW for 15 min was maintained before and after mixing. The Calisto processing program was employed for the baseline subtraction and peak interaction.

### 2.5 Computational Details

When predicting the thermochemical, vibrational, electronic, and secondary interactions of uranyl halide compounds, DFT calculations have proven to be highly effective, requiring significantly less computation time than wavefunction-based methods.^3,9,10,26^ Thus, periodic DFT was chosen as our computational method of choice. All DFT calculations were done using the Vienna Ab initio Simulation Package (VASP).^27−29^ The generalized gradient approximation of Perdew–Burker–Enrzerhof (GGA-PBE)^30^ was used to model exchange-correlation energy and projected augmented wave (PAW) pseudopotentials^31,32^ were used to represent the atoms. A plane-wave basis set cutoff of 550 eV and a γ-centered Monkhorst–Pack^33^*k*-grid of 4 × 4 × 4 was used. All structures were subjected to full geometry optimization without symmetry constraints, and the forces and total energy converged to within 1 meV Å^–1^ and 1 × 10^–8^ eV, respectively. A Hubbard *U* correction^34−36^ was applied to the uranium *f* states following the approach of Dudarev et al. with a *U*–*J* value of 4.0 eV.^37^ The van der Waals dispersion correction schemes DFT-D3 including the Becke–Johnson damping term^38^ was used in all of the DFT calculations. Vibrational calculations were performed with the finite-displacement method via the Phonopy package.^39^ Bond orders (BO) were calculated with the density-derived electrostatic and chemical 6 (DDEC6) approach implemented in the Chargemol program.^40−43^

## 3 Results and Discussion

### 3.1 Structural Description and Bond Analysis

**Figure 1:**
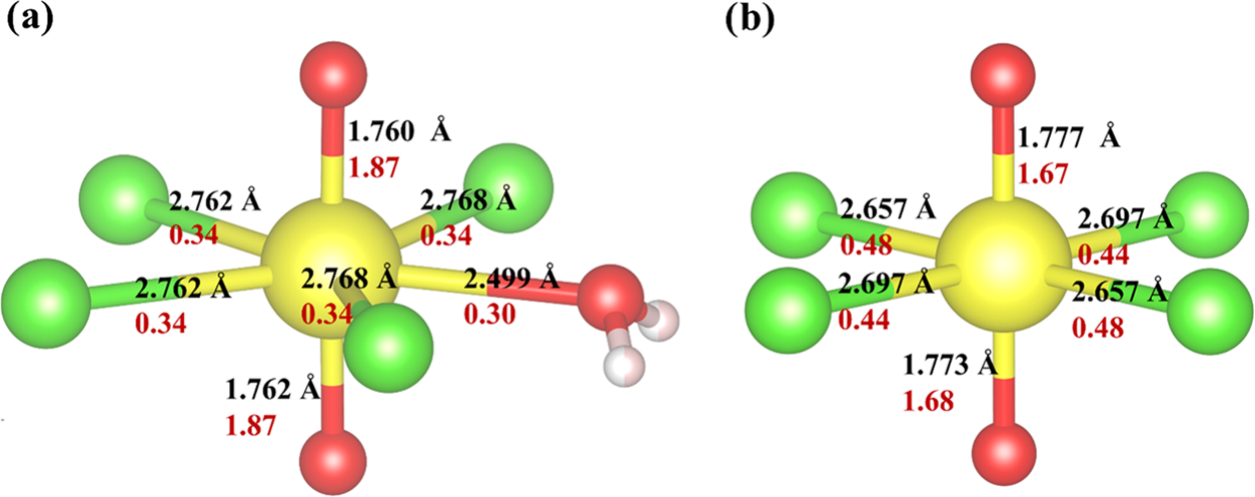
Experimental bond distances and bond orders of (a) [UO_2_Cl_4_(H_2_O)]^2–^ and (b) [UO_2_Cl_4_]^2–^. The yellow, green, red, and pink colors represent U, Cl, O, and H, respectively. The values in red indicate the calculated bond orders.

Single-crystal X-ray diffraction results identified the presence of the [UO_2_Cl_4_(H_2_O)]^2–^ unit within the (C_4_H_12_N_2_)_2_[UO_2_Cl_4_(H_2_O)]Cl_2_ solid-state compound (Figure 1a). This species contains hexavalent uranium strongly bound to two oxygen atoms to create the nearly linear dioxo cation (UO_2_^2+^). Structural analysis of the compound indicated that the average U=O bond distances are 1.761(2) Å and the O=U=O angle is 178.88 (9)°. The uranyl cation is further coordinated about the equatorial plane by four Cl^–^ anions and one ligated water molecule, with average U–Cl and U–OH_2_ distances of 2.765(5) and 2.499(5) Å, respectively. Overall, this creates a pentagonal bipyramidal coordination geometry around the U(VI) metal center.

Comparing [UO_2_Cl_4_(H_2_O)]^2–^ (Figure 1a) to that of [UO_2_Cl_4_]^2–^ (Figure 1b) allows us to consider differences in the uranyl oxo bonds and the U(VI)–Cl distances. Though the (C_4_H_12_N_2_)[UO_2_Cl_4_] structure is available, the uranyl oxo groups within the structure engage in strong axial hydrogen bonding and may not offer an accurate comparison.^9^ Thus, (C_5_H_6_N)_2_[UO_2_Cl_4_]_(s)_^1^ is taken as the reference compound to contrast the bonding differences between the [UO_2_Cl_4_(H_2_O)]^2–^ and [UO_2_Cl_4_]^2–^ complexes. With respect to experimental bond distances, the U=O bonds and U–Cl bonds of [UO_2_Cl_4_(H_2_O)]^2–^ show 0.79% contraction and 3.28% elongation, respectively, compared to [UO_2_Cl_4_]^2–^ (Figure 1). In order to rationalize the changes in U=O and U–Cl bond lengths, Bond Order (BO) analysis (SI eq 1) was performed on (C_4_H_12_N_2_)_2_[UO_2_Cl_4_(H_2_O)]Cl_2(s)_ and the reference compound using the DDEC6 method.^40−43^ Comparing BOs of [UO_2_Cl_4_(H_2_O)]^2–^ with [UO_2_Cl_4_]^2–^ indicates an ∼12% increase in the U=O BO (U=O becomes stronger) and ∼23% decrement in the U–Cl BO (U–Cl become weaker). This result agrees with the crystallographic observations of U=O bond contraction and U–Cl bond elongation, and similar strengthening of the U=O bond is reported by Cahill et al. when the pyrazine ligand directly bound to the uranyl center in [UO_2_Cl_3_(H_2_O)(pyz)_0.5_]^2–^ dimer complexes.^22^

**Figure 2:**
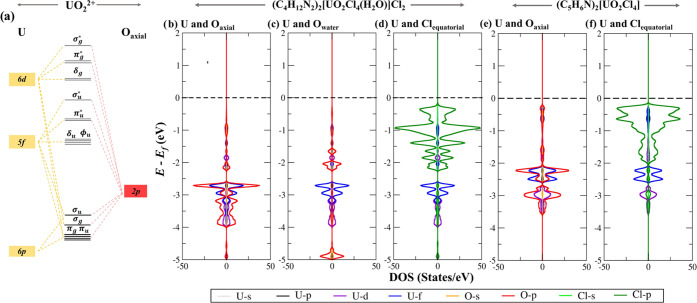
(a) Orbital mixing diagram of the uranyl cation. (b) U–O_ax_, (c) U–OH_2eq_, and (d) U–Cl_equiv_ projected density of states (PDOS) of (C_4_H_12_N_2_)_2_[UO_2_Cl_4_(H_2_O)]Cl_2(s)_. The (e) U–O_ax_ and (f) U–Cl_equiv_ projected density of states of (C_5_H_6_N)_2_[UO_2_Cl_4_]_(s)_.

To further probe changes in the primary coordination sphere, we have plotted the Projected Density of States (PDOS) for the materials containing the [UO_2_Cl_4_(H_2_O)]^2–^ or [UO_2_Cl_4_]^2–^ species (Figure 2). The electronic structure of the gas-phase uranyl cation is well-described experimentally and computationally.^10,44−46^ Sigma bonding in the uranyl cation is a result of U 6d_σ_ mixing with O 2p_σ_ and U 5f_σ_ mixing with O 2p_σ_ to form σ_g_ and σ_u_ molecular orbitals, respectively. The π_g_ orbitals are a result of U 6d_π_ and 6p_π_ blending with O 2p_π_ and the overlap of U 6p_π_ and U 5f_π_ with O 2p_π_ results in the formation of the π_u_ orbitals (Figure 2a).^45,46^ Turning to the PDOS of the [UO_2_Cl_4_(H_2_O)]^2–^ system, we noticed that the energy of U 5f, 6d, and O_axial_ 2p states had shifted −0.5 eV compared to the [UO_2_Cl_4_]^2–^ system. The σ_g_ and π_g_ of [UO_2_Cl_4_(H_2_O)]^2–^ are centered at −3.50 eV (Figure 2b), whereas σ_g_ and π_g_ of [UO_2_Cl_4_]^2–^ are centered at −3.00 eV (Figure 2c). Similarly, both σ_u_ and π_u_ of [UO_2_Cl_4_(H_2_O)]^2–^ lie ∼0.5 eV lower than [UO_2_Cl_4_]^2–^. Overall, this indicates the stabilization of U=O bonding orbitals.

The energy of Cl 2p orbitals does not differ significantly between the two systems (Figure 2b,e), and the O_water_ 2p, U 5f, and Cl 2p orbitals show densities in matching energies. This competition between O_water_ and Cl for the same 5f levels and increased steric hindrance in the equatorial plane provided an additional explanation for the lowered BOs and U–Cl bond elongation in [UO_2_Cl_4_(H_2_O)]^2^.

### 3.2 Hydrogen Bonding Within the Extended Lattice

**Figure 3:**
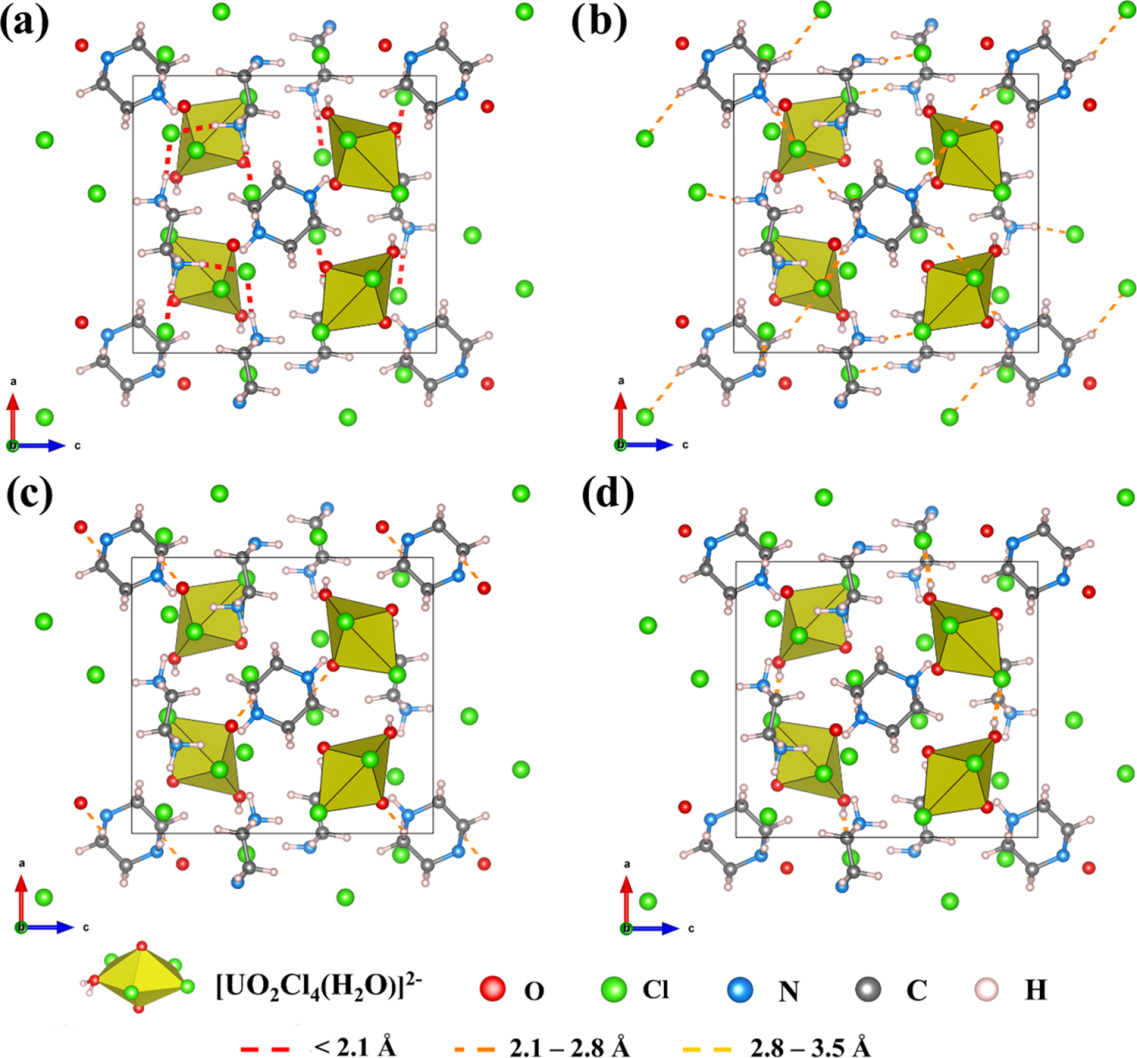
Hydrogen bond network of (C_4_H_12_N_2_)_2_[UO_2_Cl_4_(H_2_O)]Cl_2(s)_. Hydrogen bonds are shown in dashed lines, and the color of the line represents the length of the interaction. The figure shows hydrogen bonding between (a) the undercoordinated chloride ion and piperazinium cation, (b) equatorial chloride and the piperazinium cation, (c) axial oxygen and the piperazinium cation, and (d): equatorial water and equatorial chloride of neighboring [UO_2_Cl_4_(H_2_O)]^2–^ units. The VESTA^50,51^ visualization file is provided as part of the Supporting Information.

As evident from previous work, hydrogen bonding interactions are a major driver for the crystallization of solid-state materials and were analyzed in additional detail.^9,47^ The extended lattice of this compound is primarily assembled via hydrogen bonding, and previously, we devised a strategy for visualizing and quantifying the hydrogen bond network in [UO_2_X_4_]^2–^ (X = Cl^–^ or Br^–^) containing hybrid materials.^9^ Using this technique, the hydrogen bond network of (C_4_H_12_N_2_)_2_[UO_2_Cl_4_(H_2_O)]Cl_2(s)_ was successfully mapped (Figure 3) and the energetics of the interactions further analyzed. The hydrogen bonding energy (*E*_H,norm_^total^) (equations used in the *E*_H,norm_^total^ calculation are provided in the Supporting Information, SI eqs 2 and eq 3) and the number of hydrogen (*N*_H_) bonding interactions of (C_4_H_12_N_2_)_2_[UO_2_Cl_4_(H_2_O)]Cl_2(s)_ averaged per formula unit were −2404.69 kJ/mol and 39, respectively. Both *E*_H,norm_^total^ and *N*_H_ are substantially different than related (C_4_H_12_N_2_)[UO_2_Cl_4_] (*E*_H,norm_^total^ = −1394.49 kJ/mol *N*_H_ = 20).^9^ A primary reason for the more negative *E*_H,norm_^total^ for (C_4_H_12_N_2_)_2_[UO_2_Cl_4_(H_2_O)]Cl_2(s)_ is the presence of uncoordinated chloride ions in the lattice, which can act as additional hydrogen bond acceptors. These chloride anions form strong hydrogen bonds with the piperazinium cation (Cl^–^···H–N 1.989 and 1.972 Å), as evidenced by BO values of 0.199 and 0.210 (Figure 3a). The hydrogen bond network of (C_4_H_12_N_2_)_2_[UO_2_Cl_4_(H_2_O)]Cl_2(s)_ contains both A···H–N and A···H–C (A = hydrogen bond acceptor) type hydrogen bonds, which is in agreement with our previous observations.^9^ The Cl_equiv_···H–N bond distances range from 2.114 Å (BO = 0.153) to 2.683 Å (BO = 0.037), whereas the Cl_equiv_···H–C interactions are much weaker than Cl_equiv_···H–N and have BOs ranging from 0.067 to 0.020 (Figure 3b). Participation of the uranyl oxo group in hydrogen bonding is minimal, where only type O_axial_···H–C bonding is observed (2.258 Å and BO = 0.067) (Figure 3c). In contrast, the analogous (C_4_H_12_N_2_)[UO_2_Cl_4_] showed extensive hydrogen bonding through axial oxygen (BO = 0.161).^9^ Within the literature, this phenomenon is typically attributed to the reduced Lewis basicity of axial oxygen due to U=O bond strengthening^22,48,49^ and agrees with observations described in Section 3.2. The equatorial water molecule engages in strong H-bonding (Cl_equiv_···H–O_water_ 2.167 Å, BO = 0.128) with neighboring uranyl units, forming supramolecular chains of the [UO_2_Cl_4_(H_2_O)]^2–^ unit extending in the [100] direction (Figure 3d). A comprehensive list of interactions can be found in the Supporting Information, Table S4.

### 3.3 Vibrational Analysis

**Figure 4:**
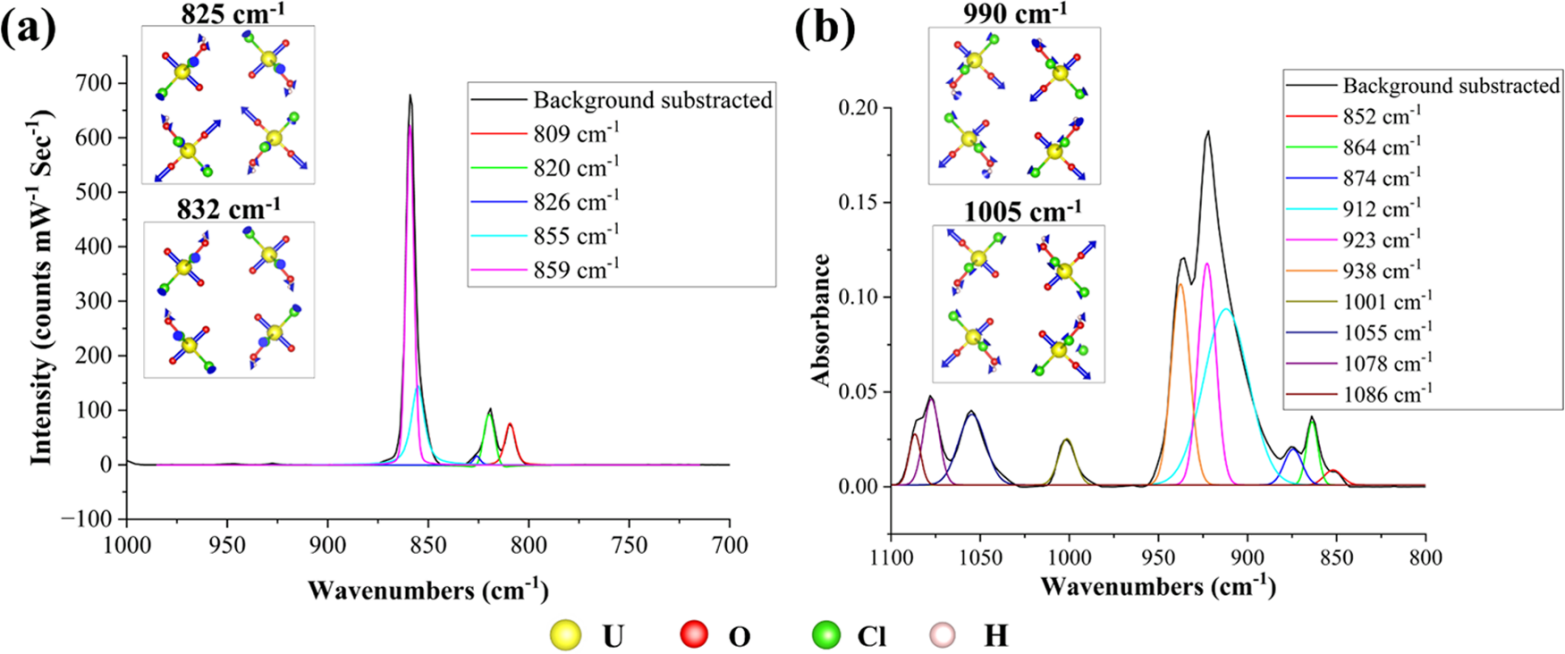
(a) Fitted Raman spectrum of (C_4_H_12_N_2_)_2_[UO_2_Cl_4_(H_2_O)]Cl_2(s)_ in the spectral window of 700–1000 cm^–1^ and the two calculated uranyl ν_1_ modes where 825 and 832 cm^–1^ corresponds to out-of-phase and in-phase uranyl vibrations. (b) Fitted IR spectrum of (C_4_H_12_N_2_)_2_[UO_2_Cl_4_(H_2_O)]Cl_2(s)_ in the spectral window of 800–1100 cm^–1^ and the two calculated uranyl ν_3_ modes where 990 and 1005 cm^–1^ corresponds to out-of-phase and in-phase uranyl vibrations.

There are three fundamental vibrational modes associated with the *D*_∞h_ symmetry uranyl cation: symmetrical stretch (ν_1_), bending mode (ν_2_), and asymmetrical stretch (ν_3_).^52,53^ The ν_1_ mode is Raman active, while the ν_2_ and ν_3_ modes are also IR active. However, owing to instrument limits and peak intensities, we are best able to track the ν_1_ and ν_3_ modes. The peak centroid for the ν_1_ of [UO_2_Cl_4_]^2–^ in solution is typically reported at 854 cm^–1^,^52,53^ but in solid-state compounds, the uranyl ν_1_ band is red-shifted to 815–845 cm^–1^ due to increased noncovalent interactions to the uranyl oxo groups.^5,6,9^ The Raman spectrum of (C_4_H_12_N_2_)_2_[UO_2_Cl_4_(H_2_O)]Cl_2(s)_ displays two distinct bands at 855 and 859 cm^–1^ within the spectral window of interest (Figure 4a) and two features (923 and 938 cm^–1^) are also noted in the IR within the region associated with the asymmetric uranyl stretching mode (Figure 4b). To investigate the origin of these double bands, we conducted computational phonon analysis utilizing the approach used by Spano et al.^54^ Here, we calculate the total displacement amplitude (*S*_yl_^n^) for the uranyl cation and phase angle between the displacement vectors of the two uranyl oxo groups (θ^n^) to distinguish Raman and IR active modes of the uranyl center. The interpretation is that a larger value of *S*_yl_^n^ accompanies a higher level of engagement of the uranyl center in normal mode. When θ^n^ is ∼180°, it indicates Raman active modes, whereas θ^n^ of ∼0° indicates IR active modes. The equations used to calculate *S*_yl_^n^ and θ^n^ are given in the Supporting Information, Section 6.3: SI eqs 4–6. Our calculations specify that the four uranyl cations in the unit cell result in two ν_1_ bands (calculated at 825 and 832 cm^–1^) and two ν_3_ bands (calculated at 990 and 1005 cm^–1^). The double signals ν_1_ and ν_3_ can be attributed to the in-phase and out-of-phase symmetrical and asymmetrical stretches associated with four unique uranyl cations in the crystalline lattice (Figure 4). All other peaks in the Raman and IR spectra can be assigned as pure or combination vibration modes of piperazinium ions. Table 2 summarizes the experimental and computational vibrational analysis. The uranyl ν_1_ and ν_3_ features of (C_4_H_12_N_2_)_2_[UO_2_Cl_4_(H_2_O)]Cl_2(s)_ are blue-shifted compared to the (C_5_H_6_N)_2_[UO_2_Cl_4_] compound (ν_1_ = 832 cm^–1^and ν_3_ = 911 cm^–1^).^9^ This provides additional proof of the strengthening of the U=O bond due to the additional water in the equatorial plane. Additionally, we determined the U=O bond length using ν_1_ and ν_3_ of (C_4_H_12_N_2_)_2_[UO_2_Cl_4_(H_2_O)]Cl_2(s)_ and (C_5_H_6_N)_2_[UO_2_Cl_4_] compounds, as defined by Wilkin, to be 1.730 and 1.739 Å.^55^ These calculated values do not agree with crystallographic obtained values; this observation agrees with our previous work, where we showed that U=O bond lengths cannot completely explain subtle shifts in uranyl vibration frequencies.^9^

**Table 2:** Summary of Experimental (exp) and Calculated (calc) Vibrational Modes of ν_1_ and ν_3_^a^

vibrational mode	exp (cm^–1^)	calc avg (cm^–1^)	calc (cm^–1^)	U1, *S*_yl_^n^	U1, θ*^n^*	U2, *S*_yl_^n^	U2, θ*^n^*	U3, *S*_yl_^n^	U3, θ*^n^*	U4, *S*_yl_^n^	U4, θ*^n^*
ν_1_	855	825	824	0.16	179.6	0.16	179.6	0.16	179.6	0.16	179.6
			826	0.12	179.6	0.12	179.6	0.12	179.6	0.12	179.6
	859	832	831	0.15	179.9	0.15	179.9	0.15	179.9	0.15	179.9
			833	0.14	179.7	0.14	179.7	0.14	179.7	0.14	179.7
ν_3_	923	990	989	0.16	0.4	0.16	0.4	0.16	0.4	0.16	0.4
			992	0.11	0.4	0.11	0.4	0.11	0.4	0.11	0.4
	938	1005	1005	0.15	0.6	0.15	0.6	0.15	0.6	0.15	0.6
			1005	0.10	0.5	0.10	0.5	0.10	0.5	0.10	0.5

a Full list of normal modes is given in the Supporting Information, Section 6.3: Table S6.

### 3.4 Formation Enthalpy (Δ*H*_f_)

The formation enthalpy of (C_4_H_12_N_2_)_2_[UO_2_Cl_4_(H_2_O)]Cl_2(s)_ was determined to assess the stability of the crystalline phase. The solvation enthalpy (Δ*H*_sol_) of (C_4_H_12_N_2_)_2_[UO_2_Cl_4_(H_2_O)]Cl_2(s)_ was determined using the isothermal acid calorimeter and then used in the thermocycle provided in Table 3 to calculate the Δ*H*_f_ of the crystalline phase. The calculated Δ*H*_f_ for (C_4_H_12_N_2_)_2_[UO_2_Cl_4_(H_2_O)]Cl_2(s)_ is −287.60 ± 1.75 kJ/mol, which indicates higher stability than the related (C_4_H_12_N_2_)[UO_2_Cl_4_]_(s)_ material (Δ*H*_f_ = −152.41 ± 1.28 kJ/mol).^9^ In previous work, we found that protonation enthalpy of the organic base and *E*_H_^total^ positively correlates with Δ*H*_f_ uranyl hybrid materials,^9^ and similar observation is noted for (C_4_H_12_N_2_)_2_[UO_2_Cl_4_(H_2_O)]Cl_2(s)_ as well. In the thermocycle used for the Δ*H*_f_ calculation, the exothermic protonation enthalpy of the organic ligand plays a significant role in the overall negative value for the reaction. Thus, the presence of two piperazinium cations in the formula unit of (C_4_H_12_N_2_)_2_[UO_2_Cl_4_(H_2_O)]Cl_2(s)_ results in a greater magnitude of Δ*H*_f_ than (C_4_H_12_N_2_)[UO_2_Cl_4_]_(s)._ The (C_4_H_12_N_2_)_2_[UO_2_Cl_4_(H_2_O)]Cl_2(s)_ compound also has a much stronger hydrogen bond network than (C_4_H_12_N_2_)[UO_2_Cl_4_]_(s)_ as evident by higher *E*_H_^total^, making it more stable as reflected in the magnitude of Δ*H*_f_.

**Table 3:** Thermocycle Used in the Calculation of Formation Enthalpies of Crystalline (C_4_H_12_N_2_)_2_[UO_2_Cl_4_(H_2_O)]Cl_2(s)_

reaction	enthalpy (kJ mol^–1^)
	–71.02^2^
	–99.57 ± 0.46^9^
	4 × 2.16^2^
	–30.40 ± 0.57
	
	–287.60 ± 1.75

Given the stability of the (C_4_H_12_N_2_)_2_[UO_2_Cl_4_(H_2_O)]Cl_2(s)_ compound compared to (C_4_H_12_N_2_)[UO_2_Cl_4_]_(s),_ it is somewhat surprising that that the aquatetrachloroU(VI) complex is not observed more often during crystallization in these systems. In this particular case, a higher ratio of the organic base is necessary to create pure yields (Supporting Information; Table S1) of (C_4_H_12_N_2_)_2_[UO_2_Cl_4_(H_2_O)]Cl_2(s)_, and that is consistent with the need for additional counterions in the solid phase. However, this suggests that by increasing the counterions in the solution, the aquatetrachloroU(VI) complex may be isolated in other systems. It is also interesting to note that [UO_2_Cl_4_(H_2_O)]^2–^ can crystalize even at very high HCl concentrations. This observation agrees with previous reports where water coordination to the uranyl center was confirmed even at high [Cl^–^] concentrations, suggesting a dynamic equilibrium between [UO_2_Cl_4_(H_2_O)]^2–^ and [UO_2_Cl_4_]^2–^ that is likely related to water availability.^12,15^

## 4 Conclusions

Synthetic conditions that result in exclusive crystallization of (C_4_H_12_N_2_)_2_[UO_2_Cl_4_(H_2_O)]Cl_2(s)_ are reported, and structural characterization was performed using single-crystal X-ray diffraction. DFT calculations in this study demonstrated that changing the primary coordination sphere from [UO_2_Cl_4_]^2–^ to [UO_2_Cl_4_(H_2_O)]^2–^ significantly strengthens the U=O bonds due to the stabilization of uranyl σ_g_, σ_u_, π_g_, and π_u_ orbitals. Stronger U=O bonding is also reflected in minimal hydrogen bonding with the oxo group and manifests in blue-shifting of the uranyl stretching features in the vibrational spectroscopy. With a stronger U=O bond, there is a reduction in the Lewis basicity of the axial oxygen atom, which limits additional hydrogen bonding interactions. Through calorimetry, the stability of the (C_4_H_12_N_2_)_2_[UO_2_Cl_4_(H_2_O)]Cl_2(s)_ crystalline phase was determined to be higher than its analogous [UO_2_Cl_4_]^2–^ compound and could be linked to the exothermic protonation enthalpy of the organic ligand and more extensive hydrogen bonding network. This study provided an in-depth understanding of water substitution within the uranyl chloride system that provides additional insights into tuning uranyl oxo interactions and the impacts of hydrogen bonding on coordination compound stability.

## Supporting Information Available

Synthesis optimization; crystallography; computational detail; bulk characterization; vibrational analysis; thermochemistry (PDF)
Piperazinium2_UO_2_Cl_4__H_2_O_Cl_2_.vesta (ZIP)
