# Supplementary material for: Synthesis, Characterization, and Density Functional Theory Investigation of the Solid-State [UO2Cl4(H2O)]2– Complex
Source: Inorg Chem. 2023 Aug 23;62(35):14318–25. doi: 10.1021/acs.inorgchem.3c01725 (PMC10481372; doi:10.1021/acs.inorgchem.3c01725)
Supplement: Supplementary file 1 — ic3c01725_si_001.pdf [file ic3c01725_si_001.pdf]

Supporting Information for: Synthesis, characterization, and Density Functional Theory investigation of solid-state $[UO_2Cl_4(H_2O)]^{2-}$ complex

Harindu Rajapaksha[a], Sara E. Mason*[a,b], Tori Z. Forbes*[a]

[a] *Department of Chemistry, University of Iowa, Iowa City, IA 52242, USA*

[b] *Center for Functional Nanomaterials, Brookhaven National Laboratory, Upton, NY 11973, USA*

*Corresponding Authors: Tori Z. Forbes Email: tori-forbes@uiowa.edu, Sara E. Mason Email: smason@bnl.gov

Table of Contents

1. Synthesis optimization....3

Table S1: The reaction mixture used in the synthesis. 3

2. Crystallography....4

Figure S1: The Thermal ellipsoid plot of $(C_4H_{12}N_2)_2[UO_2Cl_4(H_2O)]Cl_{2(s)}$ generated through CheckCIF...4

3. Computational details 4

Table S2: Comparison between experimental and theoretical lattice parameters....4

Table S3: Comparison between average experimental and theoretical bond lengths of uranyl moiety....5

3.1 Bond Oder (BO) Calculation....5

4. Hydrogen bond analysis 5

4.1 Hydrogen bond energy $(E_{H,normtotal})$....5

Table S4: The full list of hydrogen bonding interactions in the unit cell of $(C_4H_{12}N_2)_2[UO_2Cl_4(H_2O)]Cl_{2(s)}$....6

5. Bulk Characterization....8

5.1 Powder x-ray diffraction 8

Figure S2: The overlapped experimental and calculated PXRD diffractograms of $(C_4H_{12}N_2)_2[UO_2Cl_4(H_2O)]Cl_{2(s)}$, indicating the purity of the bulk crystalline phase. 8

5.2 Combustion elemental analysis 8

Table S5: The C, N and H percentages obtained by combustion elemental analysis. 8

6. Vibrational analysis....9

6.1 Raman spectroscopy....9

Figure S3: Fitted Raman spectrum of $(C_4H_{12}N_2)_2[UO_2Cl_4(H_2O)]Cl_{2(s)}$ in the spectral window of 700 – 1000 cm^{-1}. 9

6.2 IR spectroscopy 10

Figure S4: Fitted IR spectrum of $(C_4H_{12}N_2)_2[UO_2Cl_4(H_2O)]Cl_{2(s)}$ in the spectral window of 800 – 1100 cm^{-1}. 10

6.3 Computational phonon analysis 11

Table S6: All phonon mode of $(C_4H_{12}N_2)_2[UO_2Cl_4(H_2O)]Cl_{2(s)}$. The uranyl symmetric ($\nu_1$) and asymmetric ($\nu_3$) stretches are highlighted in red. 11

Figure S5: Phonon eigenvectors of normal modes centered at 825 cm^{-1}, 832 cm^{-1}, 990 cm^{-1}, and 1005 cm^{-1} are shown in figure a, b, c, and d respectively. The piperazinium cation and uncoordinated chlorides contribute slightly to the normal mode, thus they have not displayed her to make the image appear clear. 25

7. Thermochemistry 26

Table S7: Solvation enthalpies of $(C_4H_{12}N_2)_2[UO_2Cl_4(H_2O)]Cl_{2(s)}$ in 2N HCl at 25.0 ± 0.1 °C and ambient pressure 26

8. References 26

1. Synthesis optimization

Table S1: The reaction mixture used in the synthesis.

Uranyl acetate dihydrate (mg)	MeOM (mL)	12N HCl (mL)	0.4 M Piperazine (mL)	Products
120	2.00	6.00	1.00	
120	2.00	4.00	1.00	
120	2.00	2.00	1.00	
120	2.00	1.00	1.00	
120	2.00	0.80	1.00	
120	2.00	0.60	1.00	
120	2.00	0.50	1.00	
120	2.00	2.00	0.80	
120	2.00	2.00	0.40	
120	2.00	2.00	0.20	
120	2.00	1.00	2.00	
120	2.00	1.00	2.50	
120	2.00	1.00	3.00	
120	2.00	1.00	3.50	

$(C_4H_{12}N_2)_2[UO_2Cl_4(H_2O)]Cl_{2(s)}$:
$(C_4H_{12}N_2)[UO_2Cl_4]_{(s)}$:
Both:

2. Crystallography

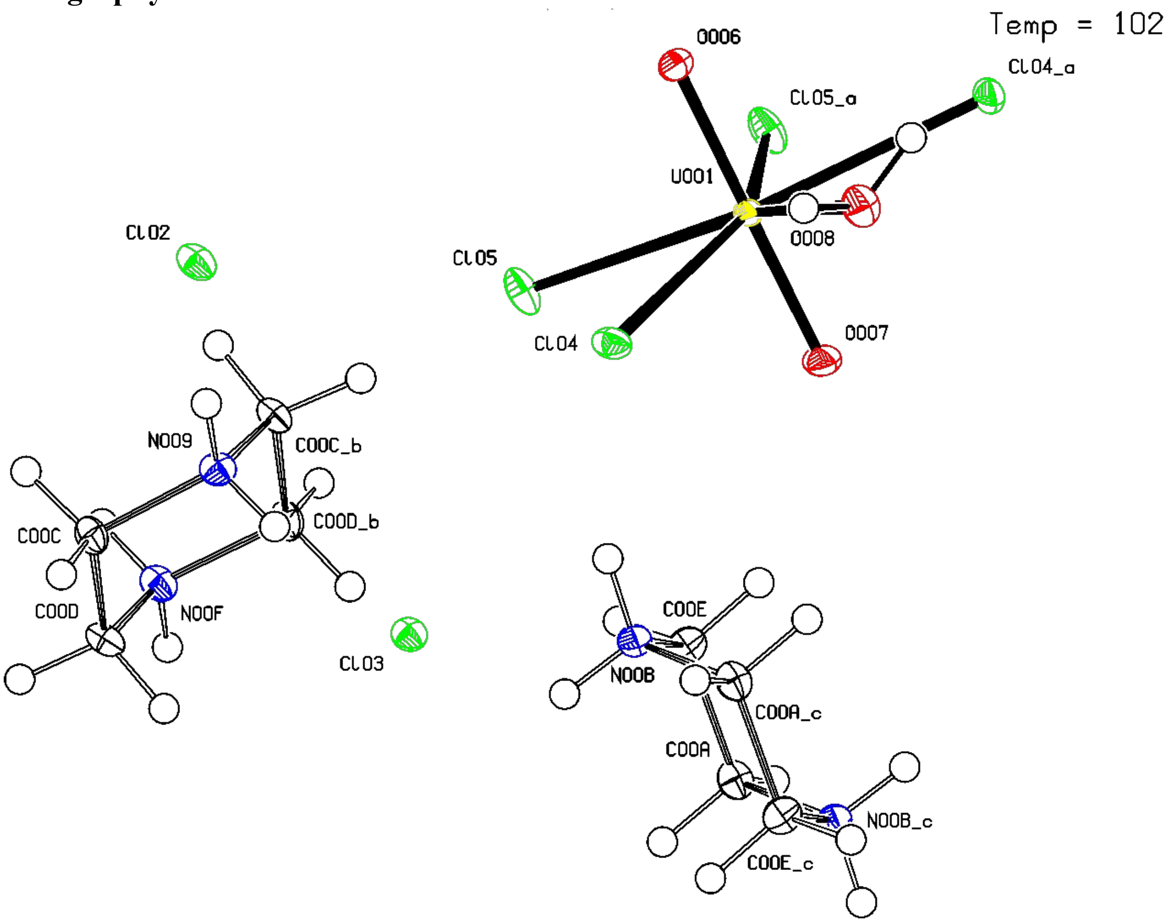

Figure S1: The Thermal ellipsoid plot of $(C_4H_{12}N_2)_2[UO_2Cl_4(H_2O)]Cl_{2(s)}$ generated through CheckCIF.

3. Computational details

The geometry optimized structure is provides as Piperazinium2_UO2Cl4_H2O_Cl2.vesta file.

Table S2: Comparison between experimental and theoretical lattice parameters

	**a (Å)**	**b (Å)**	**b (Å)**	**α (°)**	**β (°)**	**γ (°)**
**Experimental**	12.2130(7)	12.4456(7)	13.4579(8)	90°	90°	90°
**Theoretical**	12.1734	12.4730	13.3076	90°	90°	90°
**Percent Error**	-0.32 %	-0.22 %	-1.11 %	0 %	0 %	0 %

Table S3: Comparison between average experimental and theoretical bond lengths of uranyl moiety.

	**U=O (Å)**	**U-Cl (Å)**	**U-OH$_2$ (Å)**
**Experimental**	1.761(2)	2.765(5)	2.499(5)
**Theoretical**	1.770	2.798	2.515
**Percent Error**	0.51 %	1.19 %	-0.64 %

3.1 Bond Oder (BO) Calculation

The bond order between A and j atoms ($B_{A,j}$) is defined as following equation[1-4].

$$B_{A,j} = 2\oint\oint \frac{\rho_A^{\rightarrow avg}(r_A)\cdot \rho_j^{\rightarrow avg}(r'_j)}{\rho^{\rightarrow avg}(\vec{r})\cdot \rho^{\rightarrow avg}(\vec{r'})}\rho(\vec{r})\rho^{DX_{hole}}(\vec{r},\vec{r'})\, d^3\vec{r}'d^3\vec{r} \qquad \text{SI Eq 1}$$

$\rho^{\rightarrow avg}(\vec{r})$ = spherically average electron density at r

$\rho_A^{\rightarrow avg}(r_A)$ = spherically average electron density of atom A at r_A distance from A,

$\rho(\vec{r})$ = electron density at r

$\rho^{DX_{hole}}(\vec{r},\vec{r'})$ = the dressed exchange holes

4. Hydrogen bond analysis

4.1 Hydrogen bond energy ($\mathbf{E_{H,norm}^{total}}$)

$$E_{H-Bond}^{H-X,elec} = \frac{1}{4\pi\varepsilon_0}\frac{q^+q^-}{r} \qquad \text{SI Eq 2}$$

$q^+ q^-$ = Atomic charges of hydrogen and hydrogen accepter respectively

r = hydrogen interaction distance

$$E_{H,norm}^{total} = \frac{1}{Z}\sum_{i}^{H-bonds} E_{H-Bond,i}^{H-X,elec} \qquad \text{SI Eq 3}$$

Z = number of formular units

Table S4: The full list of hydrogen bonding interactions in the unit cell of $(C_4H_{12}N_2)_2[UO_2Cl_4(H_2O)]Cl_{2(s)}$.

Hydrogen atom	H accepter	Interaction distance (Å)	Bond order
41 H	13 Cl	2.698	0.0342
41 H	37 O	0.992	0.7404
41 H	24 Cl	2.167	0.1279
42 H	14 Cl	2.698	0.0342
42 H	38 O	0.992	0.7404
42 H	23 Cl	2.167	0.1279
43 H	15 Cl	2.698	0.0342
43 H	22 Cl	2.167	0.1279
43 H	39 O	0.992	0.7404
44 H	16 Cl	2.698	0.0342
44 H	21 Cl	2.167	0.1279
44 H	40 O	0.992	0.7404
45 H	17 Cl	2.698	0.0342
45 H	38 O	0.992	0.7404
45 H	28 Cl	2.167	0.1279
46 H	18 Cl	2.698	0.0342
46 H	37 O	0.992	0.7404
46 H	27 Cl	2.167	0.1279
47 H	19 Cl	2.698	0.0342
47 H	26 Cl	2.167	0.1279
47 H	40 O	0.992	0.7404
48 H	20 Cl	2.698	0.0342
48 H	25 Cl	2.167	0.1279
48 H	39 O	0.992	0.7404
49 H	5 Cl	1.989	0.1986
50 H	6 Cl	1.989	0.1986
51 H	7 Cl	1.989	0.1986
52 H	8 Cl	1.989	0.1986
53 H	9 Cl	1.972	0.21
54 H	10 Cl	1.972	0.21
55 H	11 Cl	1.972	0.21
56 H	12 Cl	1.972	0.21
57 H	8 Cl	2.810	0.0374
58 H	7 Cl	2.810	0.0374
59 H	6 Cl	2.810	0.0374
60 H	5 Cl	2.810	0.0374
61 H	7 Cl	2.810	0.0374
62 H	8 Cl	2.810	0.0374
63 H	5 Cl	2.810	0.0374
64 H	6 Cl	2.810	0.0374

Hydrogen atom	H accepter	Interaction distance (Å)	Bond order
91 H	37 O	2.626	0.0297
92 H	38 O	2.626	0.0297
93 H	40 O	2.626	0.0297
94 H	39 O	2.626	0.0297
95 H	38 O	2.626	0.0297
96 H	37 O	2.626	0.0297
97 H	18 Cl	2.770	0.0464
97 H	26 Cl	2.698	0.0466
98 H	17 Cl	2.770	0.0464
98 H	25 Cl	2.698	0.0466
99 H	20 Cl	2.770	0.0464
99 H	28 Cl	2.698	0.0466
100 H	19 Cl	2.770	0.0464
100 H	27 Cl	2.698	0.0466
101 H	14 Cl	2.770	0.0464
101 H	22 Cl	2.698	0.0466
102 H	13 Cl	2.770	0.0464
102 H	21 Cl	2.698	0.0466
103 H	16 Cl	2.770	0.0464
103 H	24 Cl	2.698	0.0466
104 H	15 Cl	2.770	0.0464
104 H	23 Cl	2.698	0.0466
105 H	8 Cl	2.792	0.0418
106 H	7 Cl	2.792	0.0418
107 H	6 Cl	2.792	0.0418
108 H	5 Cl	2.792	0.0418
109 H	7 Cl	2.792	0.0418
110 H	8 Cl	2.792	0.0418
111 H	5 Cl	2.792	0.0418
112 H	6 Cl	2.792	0.0418
113 H	19 Cl	3.176	0.0203
113 H	25 Cl	2.965	0.0272
113 H	26 Cl	2.822	0.0371
114 H	20 Cl	3.176	0.0203
114 H	25 Cl	2.822	0.0371
114 H	26 Cl	2.965	0.0272
115 H	27 Cl	2.965	0.0272
115 H	17 Cl	3.176	0.0203
115 H	28 Cl	2.822	0.0371
116 H	28 Cl	2.965	0.0272

65 H	10 Cl	2.900	0.0297
65 H	32 O	2.544	0.0357
66 H	9 Cl	2.900	0.0297
66 H	31 O	2.544	0.0357
67 H	30 O	2.544	0.0357
67 H	12 Cl	2.900	0.0297
68 H	29 O	2.544	0.0357
68 H	11 Cl	2.900	0.0297
69 H	9 Cl	2.900	0.0297
69 H	31 O	2.544	0.0357
70 H	10 Cl	2.900	0.0297
70 H	32 O	2.544	0.0357
71 H	29 O	2.544	0.0357
71 H	11 Cl	2.900	0.0297
72 H	30 O	2.544	0.0357
72 H	12 Cl	2.900	0.0297
73 H	8 Cl	2.200	0.1222
73 H	9 Cl	2.683	0.0371
74 H	7 Cl	2.200	0.1222
74 H	10 Cl	2.683	0.0371
75 H	11 Cl	2.683	0.0371
75 H	6 Cl	2.200	0.1222
76 H	12 Cl	2.683	0.0371
76 H	5 Cl	2.200	0.1222
77 H	7 Cl	2.200	0.1222
77 H	10 Cl	2.683	0.0371
78 H	8 Cl	2.200	0.1222
78 H	9 Cl	2.683	0.0371
79 H	12 Cl	2.683	0.0371
79 H	5 Cl	2.200	0.1222
80 H	11 Cl	2.683	0.0371
80 H	6 Cl	2.200	0.1222
81 H	13 Cl	2.114	0.1526
82 H	14 Cl	2.114	0.1526
83 H	15 Cl	2.114	0.1526
84 H	16 Cl	2.114	0.1526
85 H	17 Cl	2.114	0.1526
86 H	18 Cl	2.114	0.1526
87 H	19 Cl	2.114	0.1526
88 H	20 Cl	2.114	0.1526
89 H	39 O	2.626	0.0297
90 H	40 O	2.626	0.0297
116 H	18 Cl	3.176	0.0203
116 H	27 Cl	2.822	0.0371
117 H	15 Cl	3.176	0.0203
117 H	22 Cl	2.822	0.0371
117 H	21 Cl	2.965	0.0272
118 H	16 Cl	3.176	0.0203
118 H	21 Cl	2.822	0.0371
118 H	22 Cl	2.965	0.0272
119 H	13 Cl	3.176	0.0203
119 H	23 Cl	2.965	0.0272
119 H	24 Cl	2.822	0.0371
120 H	14 Cl	3.176	0.0203
120 H	24 Cl	2.965	0.0272
120 H	23 Cl	2.822	0.0371
121 H	33 O	2.258	0.0666
122 H	34 O	2.258	0.0666
123 H	35 O	2.258	0.0666
124 H	36 O	2.258	0.0666
125 H	34 O	2.258	0.0666
126 H	33 O	2.258	0.0666
127 H	36 O	2.258	0.0666
128 H	35 O	2.258	0.0666
129 H	16 Cl	2.583	0.0693
130 H	15 Cl	2.583	0.0693
131 H	14 Cl	2.583	0.0693
132 H	13 Cl	2.583	0.0693
133 H	20 Cl	2.583	0.0693
134 H	19 Cl	2.583	0.0693
135 H	18 Cl	2.583	0.0693
136 H	17 Cl	2.583	0.0693
137 H	22 Cl	2.462	0.0666
137 H	25 Cl	2.462	0.0666
138 H	21 Cl	2.462	0.0666
138 H	26 Cl	2.462	0.0666
139 H	24 Cl	2.462	0.0666
139 H	27 Cl	2.462	0.0666
140 H	23 Cl	2.462	0.0666
140 H	28 Cl	2.462	0.0666
141 H	12 Cl	1.935	0.2219
142 H	11 Cl	1.935	0.2219
143 H	10 Cl	1.935	0.2219
144 H	9 Cl	1.935	0.2219

5. Bulk Characterization

5.1 Powder x-ray diffraction

Crystals were crushed into a fine powder and put on a silicon PXRD slide with a zero background. Using a Brucke$^{r®}$ D8 advance diffractometer (CuK= 1.5406 Å) with a LynxEye detector and a Ni K-beta filter, a coupled 2 scan was performed from 5° to 60°. To determine the purity of the bulk crystalline phase, the experimental PXRD pattern was compared to the estimated PXRD pattern from a single crystal structure. Good agreement between experimental and calculated diffractograms were seen and show the purity of the crystalline phase.

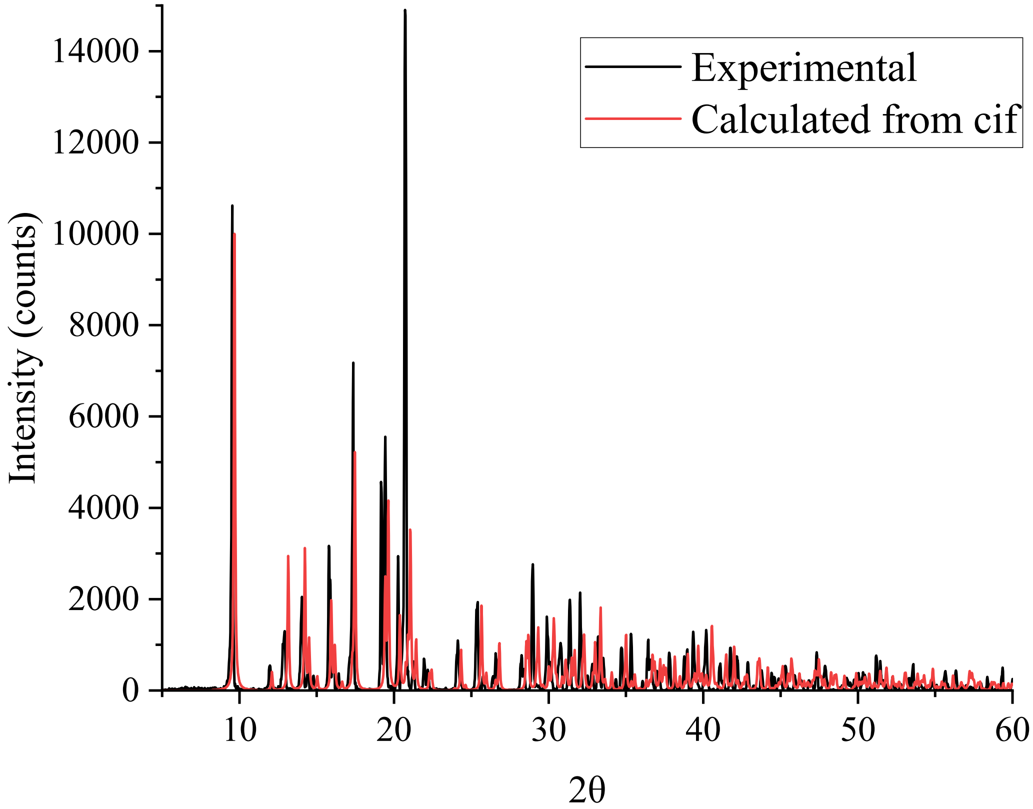

Figure S2: The overlapped experimental and calculated PXRD diffractograms of $(C_4H_{12}N_2)_2[UO_2Cl_4(H_2O)]Cl_{2(s)}$, indicating the purity of the bulk crystalline phase.

5.2 Combustion elemental analysis

The average ratios of C, N and H of the cleaned products were obtained with EAI CE-440 combustion elemental analyzer. Cysteine was used as the conditioner and standard.

Table S5: The C, N and H percentages obtained by combustion elemental analysis.

Compounds	C %	Error	N %	Error	H %	Error
$(C_4H_{12}N_2)_2[UO_2Cl_4(H_2O)]Cl_{2(s)}$	14.196	0.12 %	8.676	4.69 %	3.890	3.34 %

6. Vibrational analysis

6.1 Raman spectroscopy

Model	PsdVoigt1				
Equation	y = y0 + A * (mu * (2/pi) * (w / (4*(x-xc)^2 + w^2)) + (1 - mu) * (sqrt(4*ln(2)) / (sqrt(pi) * w)) * exp(-(4*ln(2)/w^2)*(x-xc)^2))				
Plot	Peak 1	Peak 2	Peak 3	Peak 4	Peak 5
y0	−0.70932 ± 0.2956	−0.70932 ± 0.2956	−0.70932 ± 0.2956	−0.70932 ± 0.2956	−0.70932 ± 0.2956
xc	809.25125 ± 0.3407	819.6731 ± 0.21895	825.84332 ± 0.91164	855.18474 ± 1.17762	859.07484 ± 0.05208
A	583.93897 ± 91.77286	524.57566 ± 161.93079	95.77261 ± 52.89915	1563.9599 ± 445.77042	3303.61143 ± 447.81943
w	6.10536 ± 0.32876	6.22876 ± 0.32062	4 ± 0	7.58119 ± 0.25589	4.70033 ± 0.11701
mu	0.45414 ± 0.21461	−0.55085 ± 0.94672	0.77 ± 0	0.77 ± 0	0.17341 ± 0.1422
Reduced Chi-Sqr	13.8076				
R-Square (COD)	0.99819				
Adj. R-Square	0.99807				

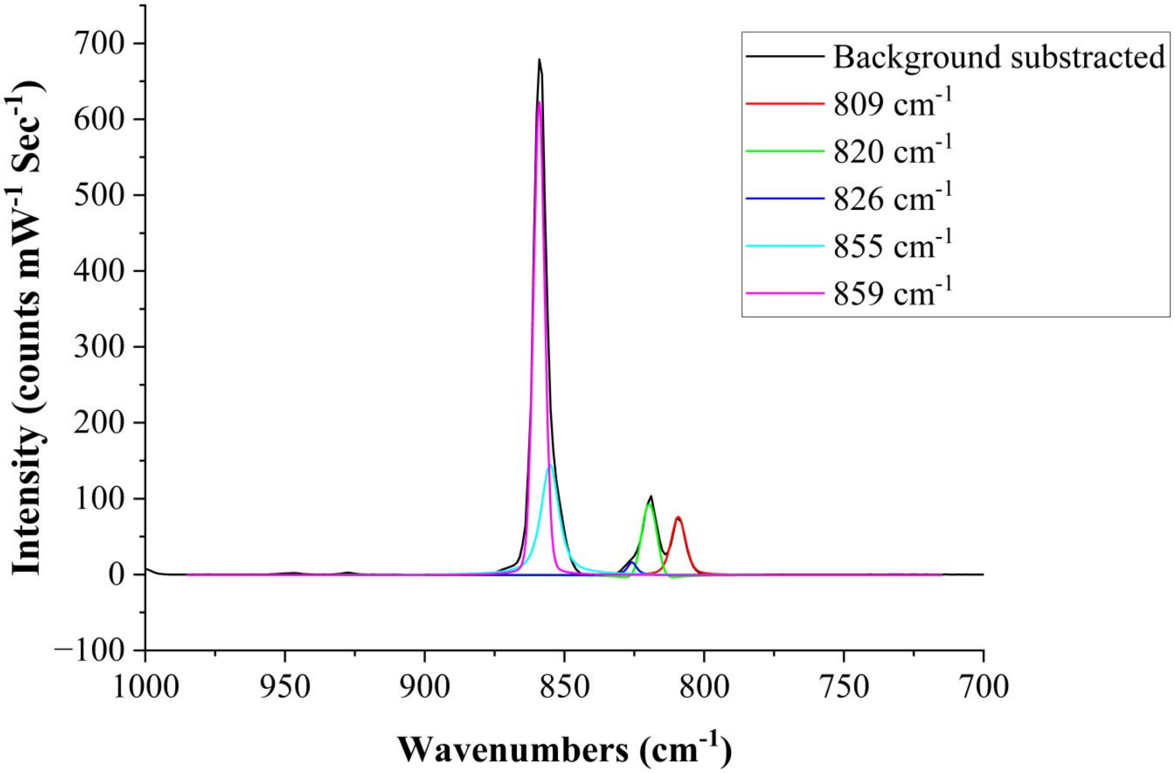

Figure S3: Fitted Raman spectrum of $(C_4H_{12}N_2)_2[UO_2Cl_4(H_2O)]Cl_{2(s)}$ in the spectral window of 700 – 1000 cm^{-1}.

6.2 IR spectroscopy

Model	Gauss									
Equatio	y=y0 + (A/(w*sqrt(pi/2)))*exp(-2*((x-xc)/w)^2)									
Plot	Peak 1	Peak 2	Peak 3	Peak 4	Peak 5	Peak 6	Peak 7	Peak 8	Peak 9	Peak 10
y0	9.24096E−4	9.24096E−4	9.24096E−4	9.24096E−4	9.24096E−4	9.24096E−4	9.24096E−4	9.24096E−4	9.24096E−4	9.24096E−4 ±
xc	852 ± 0	863.77887 ±	874.43503 ±	912.0509 ± 0.	922.75419 ±	937.59267 ±	1001.38341 ±	1054.69459 ±	1077.47958 ±	1086.68414 ±
w	9.97445 ± 2.0	6.49589 ± 0.3	10 ± 0	25 ± 0	9.93921 ± 0.2	10.43739 ± 0.	9.3974 ± 0.56	15.09042 ± 0.	8.85385 ± 0	6.56637 ± 0.4
A	0.09938 ± 0.0	0.27194 ± 0.0	0.23735 ± 0.0	2.9137 ± 0.06	1.45833 ± 0.0	1.38836 ± 0.0	0.28758 ± 0.0	0.70582 ± 0.0	0.50307 ± 0.0	0.2216 ± 0.01
Reduce	4.32424E−6									
R-Squar	0.99779									
Adj. R-S	0.99743									

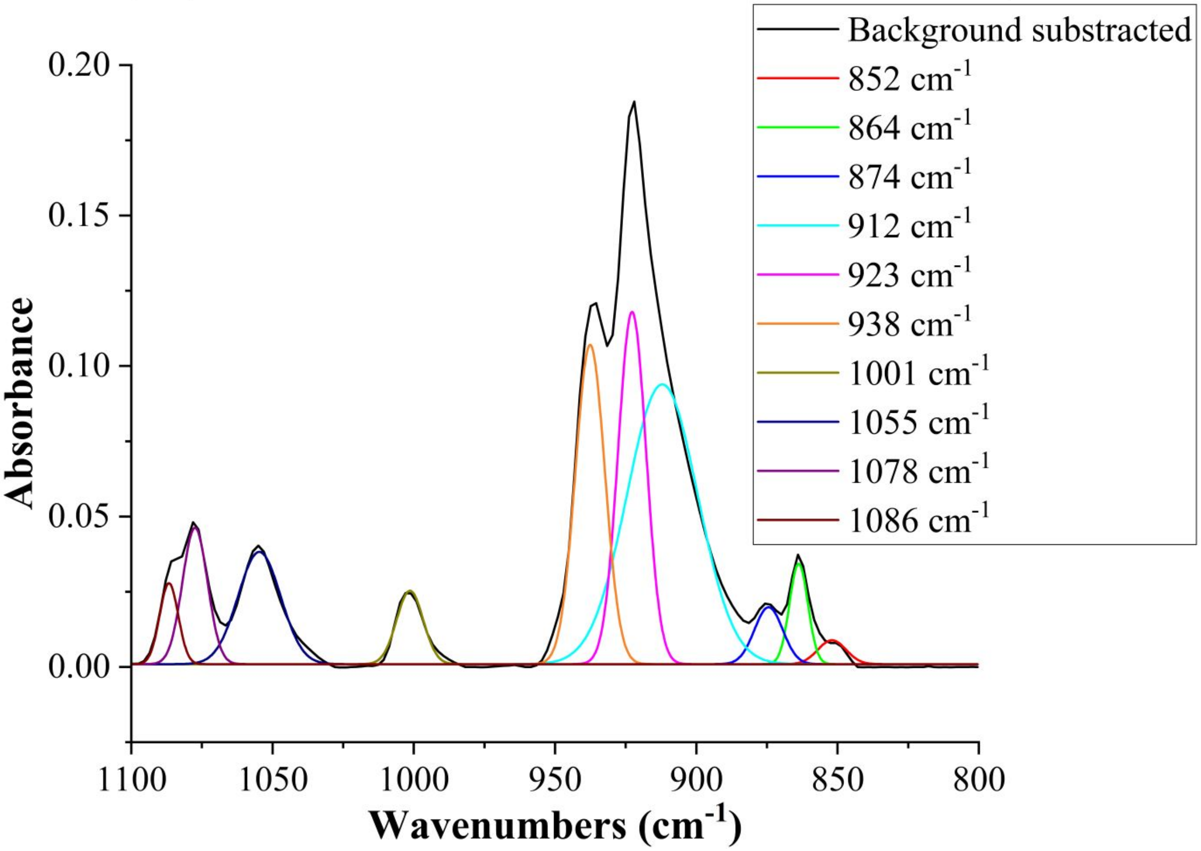

Figure S4: Fitted IR spectrum of $(C_4H_{12}N_2)_2[UO_2Cl_4(H_2O)]Cl_{2(s)}$ in the spectral window of 800 – 1100 cm^{-1}.

6.3 Computational phonon analysis

The phonon analysis is performed according to the method proposed by Spano *et al.*[5, 6]

$$S^{n}_{yl} = \sum_{i\,\in\,[yl]} |u^{n}_{i}| \qquad \text{(SI Eq 4)}$$

$$s^{n} = \frac{S^{n}_{yl}}{\sum_{i\,=\,1}^{N} |u^{n}_{i}|} \qquad \text{(SI Eq 5)}$$

$$\theta^{n} = \cos^{-1}\left(\frac{u^{n}_{oxo1} \cdot u^{n}_{oxo1}}{|u^{n}_{oxo1}| \cdot |u^{n}_{oxo1}|}\right) \qquad \text{(SI Eq 6)}$$

$|u^{n}_{i}|$ = norm of the displacement vector for each atom in the uranyl cation

S^{n}_{yl} = uranyl cation the total displacement amplitude

θ^{n} = The phase angle between the displacement vectors of the two uranyl oxo groups.

Table S6: All phonon mode of $(C_4H_{12}N_2)_2[UO_2Cl_4(H_2O)]Cl_{2(s)}$. The uranyl symmetric ($\nu_1$) and asymmetric ($\nu_3$) stretches are highlighted in red.

	**Energy (cm^{-1})**	s^n	θ^n	s^n	θ^n	s^n	θ^n	s^n	θ^n
1	-0.173	0.044	0.000	0.044	0.000	0.044	0.000	0.044	0.000
2	-0.128	0.044	0.140	0.044	0.140	0.044	0.140	0.044	0.140
3	0.010	0.044	0.085	0.044	0.085	0.044	0.085	0.044	0.085
4	22.181	0.074	1.897	0.074	1.897	0.074	1.897	0.074	1.897
5	22.508	0.061	50.327	0.061	50.327	0.061	50.327	0.061	50.327
6	25.094	0.056	0.000	0.056	0.000	0.056	0.000	0.056	0.000
7	29.019	0.059	3.476	0.059	3.476	0.059	3.476	0.059	3.476
8	34.447	0.051	0.000	0.051	0.000	0.051	0.000	0.051	0.000
9	36.968	0.024	0.000	0.024	0.000	0.024	0.000	0.024	0.000
10	37.304	0.044	148.602	0.044	148.602	0.044	148.602	0.044	148.602
11	38.104	0.070	34.155	0.070	34.155	0.070	34.155	0.070	34.155
12	38.213	0.064	0.000	0.064	0.000	0.064	0.000	0.064	0.000
13	46.850	0.031	0.000	0.031	0.000	0.031	0.000	0.031	0.000
14	47.276	0.038	70.285	0.038	70.285	0.038	70.285	0.038	70.285
15	49.624	0.013	22.504	0.013	22.504	0.013	22.504	0.013	22.504
16	49.732	0.024	0.000	0.024	0.000	0.024	0.000	0.024	0.000
17	50.206	0.021	180.000	0.021	180.000	0.021	180.000	0.021	180.000
18	52.143	0.039	169.169	0.039	169.169	0.039	169.169	0.039	169.169

19	53.058	0.050	88.203	0.050	88.203	0.050	88.203	0.050	88.203
20	56.133	0.029	127.587	0.029	127.587	0.029	127.587	0.029	127.587
21	57.084	0.022	180.000	0.022	180.000	0.022	180.000	0.022	180.000
22	58.724	0.059	8.495	0.059	8.495	0.059	8.495	0.059	8.495
23	61.110	0.020	174.316	0.020	174.316	0.020	174.316	0.020	174.316
24	61.184	0.015	103.123	0.015	103.123	0.015	103.123	0.015	103.123
25	61.980	0.015	0.000	0.015	0.000	0.015	0.000	0.015	0.000
26	62.582	0.025	180.000	0.025	180.000	0.025	180.000	0.025	180.000
27	63.285	0.034	110.614	0.034	110.614	0.034	110.614	0.034	110.614
28	64.086	0.020	180.000	0.020	180.000	0.020	180.000	0.020	180.000
29	65.195	0.048	89.138	0.048	89.138	0.048	89.138	0.048	89.138
30	66.425	0.013	0.000	0.013	0.000	0.013	0.000	0.013	0.000
31	66.662	0.032	83.888	0.032	83.888	0.032	83.888	0.032	83.888
32	68.338	0.015	180.000	0.015	180.000	0.015	180.000	0.015	180.000
33	70.159	0.042	106.250	0.042	106.250	0.042	106.250	0.042	106.250
34	71.023	0.013	0.000	0.013	0.000	0.013	0.000	0.013	0.000
35	73.021	0.022	180.000	0.022	180.000	0.022	180.000	0.022	180.000
36	73.240	0.023	61.199	0.023	61.199	0.023	61.199	0.023	61.199
37	73.603	0.024	178.346	0.024	178.346	0.024	178.346	0.024	178.346
38	75.288	0.016	180.000	0.016	180.000	0.016	180.000	0.016	180.000
39	78.356	0.017	0.000	0.017	0.000	0.017	0.000	0.017	0.000
40	79.450	0.007	180.000	0.007	180.000	0.007	180.000	0.007	180.000
41	80.490	0.010	4.992	0.010	4.992	0.010	4.992	0.010	4.992
42	80.763	0.008	180.000	0.008	180.000	0.008	180.000	0.008	180.000
43	81.222	0.020	10.865	0.020	10.865	0.020	10.865	0.020	10.865
44	81.506	0.013	180.000	0.013	180.000	0.013	180.000	0.013	180.000
45	82.445	0.023	148.165	0.023	148.165	0.023	148.165	0.023	148.165
46	83.859	0.002	180.000	0.002	180.000	0.002	180.000	0.002	180.000
47	87.289	0.009	180.000	0.009	180.000	0.009	180.000	0.009	180.000
48	87.641	0.011	112.303	0.011	112.303	0.011	112.303	0.011	112.303
49	88.134	0.012	61.567	0.012	61.567	0.012	61.567	0.012	61.567
50	88.301	0.041	74.135	0.041	74.135	0.041	74.135	0.041	74.135
51	89.027	0.003	180.000	0.003	180.000	0.003	180.000	0.003	180.000
52	91.544	0.015	0.000	0.015	0.000	0.015	0.000	0.015	0.000
53	91.650	0.023	0.000	0.023	0.000	0.023	0.000	0.023	0.000
54	92.450	0.019	114.035	0.019	114.035	0.019	114.035	0.019	114.035
55	94.068	0.013	180.000	0.013	180.000	0.013	180.000	0.013	180.000
56	94.537	0.014	180.000	0.014	180.000	0.014	180.000	0.014	180.000
57	96.750	0.005	0.000	0.005	0.000	0.005	0.000	0.005	0.000
58	97.182	0.013	0.000	0.013	0.000	0.013	0.000	0.013	0.000
59	97.307	0.017	0.000	0.017	0.000	0.017	0.000	0.017	0.000
60	97.644	0.012	180.000	0.012	180.000	0.012	180.000	0.012	180.000
61	97.665	0.012	44.137	0.012	44.137	0.012	44.137	0.012	44.137

62	100.677	0.015	180.000	0.015	180.000	0.015	180.000	0.015	180.000
63	101.056	0.012	180.000	0.012	180.000	0.012	180.000	0.012	180.000
64	101.871	0.023	98.249	0.023	98.249	0.023	98.249	0.023	98.249
65	102.609	0.007	0.000	0.007	0.000	0.007	0.000	0.007	0.000
66	102.864	0.001	180.000	0.001	180.000	0.001	180.000	0.001	180.000
67	105.729	0.024	45.680	0.024	45.680	0.024	45.680	0.024	45.680
68	105.987	0.037	11.325	0.037	11.325	0.037	11.325	0.037	11.325
69	107.096	0.030	2.965	0.030	2.965	0.030	2.965	0.030	2.965
70	108.077	0.014	0.387	0.014	0.387	0.014	0.387	0.014	0.387
71	110.464	0.012	180.000	0.012	180.000	0.012	180.000	0.012	180.000
72	110.478	0.015	180.000	0.015	180.000	0.015	180.000	0.015	180.000
73	113.930	0.008	180.000	0.008	180.000	0.008	180.000	0.008	180.000
74	114.716	0.035	71.016	0.035	71.016	0.035	71.016	0.035	71.016
75	115.813	0.019	180.000	0.019	180.000	0.019	180.000	0.019	180.000
76	116.214	0.019	93.707	0.019	93.707	0.019	93.707	0.019	93.707
77	117.110	0.030	108.233	0.030	108.233	0.030	108.233	0.030	108.233
78	120.302	0.012	157.822	0.012	157.822	0.012	157.822	0.012	157.822
79	121.747	0.013	58.305	0.013	58.305	0.013	58.305	0.013	58.305
80	125.558	0.022	175.259	0.022	175.259	0.022	175.259	0.022	175.259
81	129.411	0.058	154.225	0.058	154.225	0.058	154.225	0.058	154.225
82	130.666	0.014	62.367	0.014	62.367	0.014	62.367	0.014	62.367
83	131.276	0.042	131.623	0.042	131.623	0.042	131.623	0.042	131.623
84	131.970	0.022	180.000	0.022	180.000	0.022	180.000	0.022	180.000
85	133.681	0.023	118.257	0.023	118.257	0.023	118.257	0.023	118.257
86	134.685	0.016	33.828	0.016	33.828	0.016	33.828	0.016	33.828
87	134.789	0.023	0.000	0.023	0.000	0.023	0.000	0.023	0.000
88	135.646	0.022	176.319	0.022	176.319	0.022	176.319	0.022	176.319
89	138.259	0.028	180.000	0.028	180.000	0.028	180.000	0.028	180.000
90	139.984	0.035	180.000	0.035	180.000	0.035	180.000	0.035	180.000
91	141.185	0.024	180.000	0.024	180.000	0.024	180.000	0.024	180.000
92	148.309	0.027	148.908	0.027	148.908	0.027	148.908	0.027	148.908
93	148.797	0.034	180.000	0.034	180.000	0.034	180.000	0.034	180.000
94	149.525	0.027	154.920	0.027	154.920	0.027	154.920	0.027	154.920
95	159.566	0.012	180.000	0.012	180.000	0.012	180.000	0.012	180.000
96	160.125	0.009	180.000	0.009	180.000	0.009	180.000	0.009	180.000
97	162.443	0.019	180.000	0.019	180.000	0.019	180.000	0.019	180.000
98	162.493	0.029	165.193	0.029	165.193	0.029	165.193	0.029	165.193
99	163.173	0.010	180.000	0.010	180.000	0.010	180.000	0.010	180.000
100	163.879	0.015	152.664	0.015	152.664	0.015	152.664	0.015	152.664
101	164.326	0.007	180.000	0.007	180.000	0.007	180.000	0.007	180.000
102	165.847	0.021	180.000	0.021	180.000	0.021	180.000	0.021	180.000
103	165.915	0.026	170.300	0.026	170.300	0.026	170.300	0.026	170.300
104	167.275	0.014	147.697	0.014	147.697	0.014	147.697	0.014	147.697

105	168.626	0.009	140.882	0.009	140.882	0.009	140.882	0.009	140.882
106	171.826	0.010	128.797	0.010	128.797	0.010	128.797	0.010	128.797
107	176.337	0.011	170.278	0.011	170.278	0.011	170.278	0.011	170.278
108	177.142	0.024	177.854	0.024	177.854	0.024	177.854	0.024	177.854
109	177.589	0.018	180.000	0.018	180.000	0.018	180.000	0.018	180.000
110	180.105	0.022	179.498	0.022	179.498	0.022	179.498	0.022	179.498
111	180.957	0.017	180.000	0.017	180.000	0.017	180.000	0.017	180.000
112	181.426	0.010	180.000	0.010	180.000	0.010	180.000	0.010	180.000
113	182.088	0.009	180.000	0.009	180.000	0.009	180.000	0.009	180.000
114	184.051	0.014	171.503	0.014	171.503	0.014	171.503	0.014	171.503
115	184.570	0.025	179.905	0.025	179.905	0.025	179.905	0.025	179.905
116	185.582	0.015	171.304	0.015	171.304	0.015	171.304	0.015	171.304
117	188.622	0.012	177.182	0.012	177.182	0.012	177.182	0.012	177.182
118	189.753	0.011	162.014	0.011	162.014	0.011	162.014	0.011	162.014
119	192.401	0.015	174.677	0.015	174.677	0.015	174.677	0.015	174.677
120	192.710	0.011	178.951	0.011	178.951	0.011	178.951	0.011	178.951
121	195.423	0.051	180.000	0.051	180.000	0.051	180.000	0.051	180.000
122	197.084	0.019	166.046	0.019	166.046	0.019	166.046	0.019	166.046
123	197.137	0.051	180.000	0.051	180.000	0.051	180.000	0.051	180.000
124	197.182	0.027	170.587	0.027	170.587	0.027	170.587	0.027	170.587
125	198.497	0.063	175.376	0.063	175.376	0.063	175.376	0.063	175.376
126	200.318	0.062	180.000	0.062	180.000	0.062	180.000	0.062	180.000
127	200.410	0.069	174.649	0.069	174.649	0.069	174.649	0.069	174.649
128	202.784	0.084	180.000	0.084	180.000	0.084	180.000	0.084	180.000
129	205.295	0.020	18.309	0.020	18.309	0.020	18.309	0.020	18.309
130	206.540	0.069	175.174	0.069	175.174	0.069	175.174	0.069	175.174
131	206.642	0.066	175.339	0.066	175.339	0.066	175.339	0.066	175.339
132	210.951	0.022	12.943	0.022	12.943	0.022	12.943	0.022	12.943
133	211.947	0.078	180.000	0.078	180.000	0.078	180.000	0.078	180.000
134	212.781	0.110	180.000	0.110	180.000	0.110	180.000	0.110	180.000
135	213.501	0.067	180.000	0.067	180.000	0.067	180.000	0.067	180.000
136	213.966	0.092	180.000	0.092	180.000	0.092	180.000	0.092	180.000
137	214.714	0.030	174.430	0.030	174.430	0.030	174.430	0.030	174.430
138	215.636	0.025	175.495	0.025	175.495	0.025	175.495	0.025	175.495
139	220.932	0.019	175.060	0.019	175.060	0.019	175.060	0.019	175.060
140	221.190	0.017	175.349	0.017	175.349	0.017	175.349	0.017	175.349
141	227.820	0.021	54.253	0.021	54.253	0.021	54.253	0.021	54.253
142	228.072	0.019	165.440	0.019	165.440	0.019	165.440	0.019	165.440
143	229.303	0.024	161.976	0.024	161.976	0.024	161.976	0.024	161.976
144	230.841	0.029	37.082	0.029	37.082	0.029	37.082	0.029	37.082
145	240.452	0.007	180.000	0.007	180.000	0.007	180.000	0.007	180.000
146	240.908	0.012	180.000	0.012	180.000	0.012	180.000	0.012	180.000
147	243.858	0.002	130.317	0.002	130.317	0.002	130.317	0.002	130.317

148	244.562	0.003	6.537	0.003	6.537	0.003	6.537	0.003	6.537
149	247.553	0.003	0.000	0.003	0.000	0.003	0.000	0.003	0.000
150	248.014	0.004	0.000	0.004	0.000	0.004	0.000	0.004	0.000
151	248.033	0.001	180.000	0.001	180.000	0.001	180.000	0.001	180.000
152	248.670	0.001	0.000	0.001	0.000	0.001	0.000	0.001	0.000
153	251.191	0.009	180.000	0.009	180.000	0.009	180.000	0.009	180.000
154	251.256	0.012	180.000	0.012	180.000	0.012	180.000	0.012	180.000
155	253.974	0.009	0.000	0.009	0.000	0.009	0.000	0.009	0.000
156	254.760	0.012	0.000	0.012	0.000	0.012	0.000	0.012	0.000
157	275.613	0.012	0.000	0.012	0.000	0.012	0.000	0.012	0.000
158	276.847	0.007	0.000	0.007	0.000	0.007	0.000	0.007	0.000
159	285.650	0.003	20.411	0.003	20.411	0.003	20.411	0.003	20.411
160	287.264	0.005	11.678	0.005	11.678	0.005	11.678	0.005	11.678
161	307.178	0.004	1.245	0.004	1.245	0.004	1.245	0.004	1.245
162	307.563	0.004	0.044	0.004	0.044	0.004	0.044	0.004	0.044
163	310.805	0.006	1.095	0.006	1.095	0.006	1.095	0.006	1.095
164	311.077	0.007	0.766	0.007	0.766	0.007	0.766	0.007	0.766
165	328.678	0.134	2.154	0.134	2.154	0.134	2.154	0.134	2.154
166	328.860	0.129	2.251	0.129	2.251	0.129	2.251	0.129	2.251
167	332.612	0.119	2.026	0.119	2.026	0.119	2.026	0.119	2.026
168	332.624	0.114	2.267	0.114	2.267	0.114	2.267	0.114	2.267
169	343.183	0.172	0.000	0.172	0.000	0.172	0.000	0.172	0.000
170	344.225	0.176	0.000	0.176	0.000	0.176	0.000	0.176	0.000
171	347.912	0.170	0.000	0.170	0.000	0.170	0.000	0.170	0.000
172	349.656	0.175	0.000	0.175	0.000	0.175	0.000	0.175	0.000
173	351.035	0.089	3.098	0.089	3.098	0.089	3.098	0.089	3.098
174	351.217	0.086	6.428	0.086	6.428	0.086	6.428	0.086	6.428
175	351.645	0.096	2.930	0.096	2.930	0.096	2.930	0.096	2.930
176	351.913	0.083	6.420	0.083	6.420	0.083	6.420	0.083	6.420
177	386.165	0.008	0.000	0.008	0.000	0.008	0.000	0.008	0.000
178	386.243	0.005	0.000	0.005	0.000	0.005	0.000	0.005	0.000
179	392.936	0.001	43.329	0.001	43.329	0.001	43.329	0.001	43.329
180	393.259	0.001	106.628	0.001	106.628	0.001	106.628	0.001	106.628
181	397.082	0.002	14.331	0.002	14.331	0.002	14.331	0.002	14.331
182	397.612	0.003	0.900	0.003	0.900	0.003	0.900	0.003	0.900
183	398.411	0.001	22.757	0.001	22.757	0.001	22.757	0.001	22.757
184	398.838	0.002	36.222	0.002	36.222	0.002	36.222	0.002	36.222
185	435.240	0.004	0.000	0.004	0.000	0.004	0.000	0.004	0.000
186	436.048	0.006	0.000	0.006	0.000	0.006	0.000	0.006	0.000
187	439.931	0.011	0.000	0.011	0.000	0.011	0.000	0.011	0.000
188	443.626	0.001	135.192	0.001	135.192	0.001	135.192	0.001	135.192
189	444.800	0.008	0.000	0.008	0.000	0.008	0.000	0.008	0.000
190	444.884	0.017	0.000	0.017	0.000	0.017	0.000	0.017	0.000

191	446.176	0.002	70.974	0.002	70.974	0.002	70.974	0.002	70.974
192	448.622	0.012	0.000	0.012	0.000	0.012	0.000	0.012	0.000
193	456.619	0.001	0.000	0.001	0.000	0.001	0.000	0.001	0.000
194	457.040	0.001	0.000	0.001	0.000	0.001	0.000	0.001	0.000
195	457.441	0.002	0.000	0.002	0.000	0.002	0.000	0.002	0.000
196	458.875	0.006	0.000	0.006	0.000	0.006	0.000	0.006	0.000
197	459.535	0.004	0.000	0.004	0.000	0.004	0.000	0.004	0.000
198	459.984	0.002	0.000	0.002	0.000	0.002	0.000	0.002	0.000
199	460.323	0.002	32.214	0.002	32.214	0.002	32.214	0.002	32.214
200	460.816	0.001	163.656	0.001	163.656	0.001	163.656	0.001	163.656
201	469.541	0.002	36.616	0.002	36.616	0.002	36.616	0.002	36.616
202	470.275	0.001	107.391	0.001	107.391	0.001	107.391	0.001	107.391
203	470.504	0.001	24.711	0.001	24.711	0.001	24.711	0.001	24.711
204	471.443	0.002	11.852	0.002	11.852	0.002	11.852	0.002	11.852
205	557.560	0.003	0.000	0.003	0.000	0.003	0.000	0.003	0.000
206	558.246	0.003	0.000	0.003	0.000	0.003	0.000	0.003	0.000
207	563.039	0.001	21.874	0.001	21.874	0.001	21.874	0.001	21.874
208	564.301	0.002	162.086	0.002	162.086	0.002	162.086	0.002	162.086
209	574.955	0.001	100.888	0.001	100.888	0.001	100.888	0.001	100.888
210	575.546	0.001	95.106	0.001	95.106	0.001	95.106	0.001	95.106
211	577.082	0.001	24.312	0.001	24.312	0.001	24.312	0.001	24.312
212	577.630	0.001	27.942	0.001	27.942	0.001	27.942	0.001	27.942
213	692.736	0.004	0.000	0.004	0.000	0.004	0.000	0.004	0.000
214	693.218	0.004	0.000	0.004	0.000	0.004	0.000	0.004	0.000
215	694.190	0.007	0.000	0.007	0.000	0.007	0.000	0.007	0.000
216	695.042	0.006	0.000	0.006	0.000	0.006	0.000	0.006	0.000
217	758.988	0.022	162.244	0.022	162.244	0.022	162.244	0.022	162.244
218	759.139	0.015	87.130	0.015	87.130	0.015	87.130	0.015	87.130
219	759.605	0.019	158.086	0.019	158.086	0.019	158.086	0.019	158.086
220	760.589	0.015	109.553	0.015	109.553	0.015	109.553	0.015	109.553
221	807.725	0.000	180.000	0.000	180.000	0.000	180.000	0.000	180.000
222	807.876	0.001	0.000	0.001	0.000	0.001	0.000	0.001	0.000
223	807.913	0.012	179.461	0.012	179.461	0.012	179.461	0.012	179.461
224	808.049	0.011	178.523	0.011	178.523	0.011	178.523	0.011	178.523
225	809.032	0.000	0.000	0.000	0.000	0.000	0.000	0.000	0.000
226	809.436	0.000	180.000	0.000	180.000	0.000	180.000	0.000	180.000
227	809.831	0.016	177.462	0.016	177.462	0.016	177.462	0.016	177.462
228	809.872	0.007	175.584	0.007	175.584	0.007	175.584	0.007	175.584
229	812.755	0.000	0.000	0.000	0.000	0.000	0.000	0.000	0.000
230	812.878	0.000	180.000	0.000	180.000	0.000	180.000	0.000	180.000
231	813.665	0.001	0.000	0.001	0.000	0.001	0.000	0.001	0.000
232	813.777	0.000	0.000	0.000	0.000	0.000	0.000	0.000	0.000
233	818.106	0.003	179.176	0.003	179.176	0.003	179.176	0.003	179.176

234	818.557	0.011	179.544	0.011	179.544	0.011	179.544	0.011	179.544
235	818.592	0.008	178.844	0.008	178.844	0.008	178.844	0.008	178.844
236	818.902	0.008	179.229	0.008	179.229	0.008	179.229	0.008	179.229
237	824.118	0.161	179.562	0.161	179.562	0.161	179.562	0.161	179.562
238	825.740	0.118	179.641	0.118	179.641	0.118	179.641	0.118	179.641
239	830.706	0.145	179.872	0.145	179.872	0.145	179.872	0.145	179.872
240	832.685	0.145	179.749	0.145	179.749	0.145	179.749	0.145	179.749
241	863.818	0.000	0.000	0.000	0.000	0.000	0.000	0.000	0.000
242	863.886	0.000	180.000	0.000	180.000	0.000	180.000	0.000	180.000
243	864.218	0.001	0.000	0.001	0.000	0.001	0.000	0.001	0.000
244	864.222	0.003	179.030	0.003	179.030	0.003	179.030	0.003	179.030
245	864.304	0.001	0.000	0.001	0.000	0.001	0.000	0.001	0.000
246	864.492	0.004	179.875	0.004	179.875	0.004	179.875	0.004	179.875
247	866.170	0.001	3.102	0.001	3.102	0.001	3.102	0.001	3.102
248	866.222	0.003	179.665	0.003	179.665	0.003	179.665	0.003	179.665
249	872.386	0.000	0.000	0.000	0.000	0.000	0.000	0.000	0.000
250	872.780	0.000	180.000	0.000	180.000	0.000	180.000	0.000	180.000
251	872.927	0.006	179.289	0.006	179.289	0.006	179.289	0.006	179.289
252	873.271	0.003	4.636	0.003	4.636	0.003	4.636	0.003	4.636
253	874.762	0.001	179.519	0.001	179.519	0.001	179.519	0.001	179.519
254	874.792	0.000	0.000	0.000	0.000	0.000	0.000	0.000	0.000
255	874.824	0.000	180.000	0.000	180.000	0.000	180.000	0.000	180.000
256	875.127	0.002	3.555	0.002	3.555	0.002	3.555	0.002	3.555
257	875.183	0.000	0.000	0.000	0.000	0.000	0.000	0.000	0.000
258	875.381	0.000	0.000	0.000	0.000	0.000	0.000	0.000	0.000
259	875.921	0.000	177.083	0.000	177.083	0.000	177.083	0.000	177.083
260	876.197	0.001	177.086	0.001	177.086	0.001	177.086	0.001	177.086
261	880.549	0.001	2.716	0.001	2.716	0.001	2.716	0.001	2.716
262	880.687	0.002	163.808	0.002	163.808	0.002	163.808	0.002	163.808
263	882.433	0.001	176.593	0.001	176.593	0.001	176.593	0.001	176.593
264	882.517	0.001	178.822	0.001	178.822	0.001	178.822	0.001	178.822
265	933.553	0.000	0.000	0.000	0.000	0.000	0.000	0.000	0.000
266	933.648	0.000	0.000	0.000	0.000	0.000	0.000	0.000	0.000
267	934.365	0.015	0.197	0.015	0.197	0.015	0.197	0.015	0.197
268	936.368	0.003	135.229	0.003	135.229	0.003	135.229	0.003	135.229
269	946.093	0.001	164.681	0.001	164.681	0.001	164.681	0.001	164.681
270	946.348	0.002	173.888	0.002	173.888	0.002	173.888	0.002	173.888
271	948.033	0.001	17.171	0.001	17.171	0.001	17.171	0.001	17.171
272	948.176	0.001	30.045	0.001	30.045	0.001	30.045	0.001	30.045
273	978.419	0.000	0.000	0.000	0.000	0.000	0.000	0.000	0.000
274	978.932	0.001	0.000	0.001	0.000	0.001	0.000	0.001	0.000
275	979.929	0.000	0.000	0.000	0.000	0.000	0.000	0.000	0.000
276	979.964	0.000	0.000	0.000	0.000	0.000	0.000	0.000	0.000

277	985.873	0.041	0.761	0.041	0.761	0.041	0.761	0.041	0.761
278	985.974	0.000	0.000	0.000	0.000	0.000	0.000	0.000	0.000
279	986.048	0.000	180.000	0.000	180.000	0.000	180.000	0.000	180.000
280	988.821	0.165	0.404	0.165	0.404	0.165	0.404	0.165	0.404
281	989.640	0.036	0.758	0.036	0.758	0.036	0.758	0.036	0.758
282	992.041	0.112	0.375	0.112	0.375	0.112	0.375	0.112	0.375
283	1005.325	0.153	0.571	0.153	0.571	0.153	0.571	0.153	0.571
284	1005.496	0.102	0.455	0.102	0.455	0.102	0.455	0.102	0.455
285	1030.276	0.000	0.000	0.000	0.000	0.000	0.000	0.000	0.000
286	1031.343	0.009	1.319	0.009	1.319	0.009	1.319	0.009	1.319
287	1031.555	0.007	0.281	0.007	0.281	0.007	0.281	0.007	0.281
288	1031.821	0.001	180.000	0.001	180.000	0.001	180.000	0.001	180.000
289	1031.847	0.001	160.231	0.001	160.231	0.001	160.231	0.001	160.231
290	1032.552	0.000	143.180	0.000	143.180	0.000	143.180	0.000	143.180
291	1033.442	0.009	0.714	0.009	0.714	0.009	0.714	0.009	0.714
292	1035.059	0.013	0.215	0.013	0.215	0.013	0.215	0.013	0.215
293	1045.259	0.001	7.088	0.001	7.088	0.001	7.088	0.001	7.088
294	1045.495	0.001	4.086	0.001	4.086	0.001	4.086	0.001	4.086
295	1046.565	0.002	4.172	0.002	4.172	0.002	4.172	0.002	4.172
296	1047.024	0.002	1.250	0.002	1.250	0.002	1.250	0.002	1.250
297	1047.156	0.000	180.000	0.000	180.000	0.000	180.000	0.000	180.000
298	1047.215	0.000	180.000	0.000	180.000	0.000	180.000	0.000	180.000
299	1049.883	0.005	0.148	0.005	0.148	0.005	0.148	0.005	0.148
300	1050.068	0.003	0.726	0.003	0.726	0.003	0.726	0.003	0.726
301	1055.097	0.000	0.000	0.000	0.000	0.000	0.000	0.000	0.000
302	1055.215	0.000	180.000	0.000	180.000	0.000	180.000	0.000	180.000
303	1056.713	0.000	0.000	0.000	0.000	0.000	0.000	0.000	0.000
304	1056.782	0.000	0.000	0.000	0.000	0.000	0.000	0.000	0.000
305	1056.970	0.000	0.000	0.000	0.000	0.000	0.000	0.000	0.000
306	1056.992	0.000	0.000	0.000	0.000	0.000	0.000	0.000	0.000
307	1057.417	0.003	13.069	0.003	13.069	0.003	13.069	0.003	13.069
308	1057.953	0.008	3.110	0.008	3.110	0.008	3.110	0.008	3.110
309	1074.060	0.000	0.000	0.000	0.000	0.000	0.000	0.000	0.000
310	1074.231	0.000	180.000	0.000	180.000	0.000	180.000	0.000	180.000
311	1074.898	0.006	2.520	0.006	2.520	0.006	2.520	0.006	2.520
312	1074.930	0.004	2.925	0.004	2.925	0.004	2.925	0.004	2.925
313	1081.754	0.000	0.000	0.000	0.000	0.000	0.000	0.000	0.000
314	1081.851	0.000	0.000	0.000	0.000	0.000	0.000	0.000	0.000
315	1082.083	0.000	0.000	0.000	0.000	0.000	0.000	0.000	0.000
316	1082.117	0.000	0.000	0.000	0.000	0.000	0.000	0.000	0.000
317	1127.829	0.000	0.000	0.000	0.000	0.000	0.000	0.000	0.000
318	1127.850	0.000	0.000	0.000	0.000	0.000	0.000	0.000	0.000
319	1128.567	0.000	0.000	0.000	0.000	0.000	0.000	0.000	0.000

320	1128.896	0.000	0.000	0.000	0.000	0.000	0.000	0.000	0.000
321	1135.373	0.000	0.000	0.000	0.000	0.000	0.000	0.000	0.000
322	1136.062	0.000	0.000	0.000	0.000	0.000	0.000	0.000	0.000
323	1138.522	0.004	2.757	0.004	2.757	0.004	2.757	0.004	2.757
324	1138.960	0.001	12.759	0.001	12.759	0.001	12.759	0.001	12.759
325	1180.497	0.000	10.313	0.000	10.313	0.000	10.313	0.000	10.313
326	1181.980	0.000	173.142	0.000	173.142	0.000	173.142	0.000	173.142
327	1183.536	0.002	1.262	0.002	1.262	0.002	1.262	0.002	1.262
328	1183.997	0.001	14.151	0.001	14.151	0.001	14.151	0.001	14.151
329	1192.249	0.000	0.000	0.000	0.000	0.000	0.000	0.000	0.000
330	1192.947	0.000	180.000	0.000	180.000	0.000	180.000	0.000	180.000
331	1192.977	0.000	0.000	0.000	0.000	0.000	0.000	0.000	0.000
332	1193.118	0.000	0.000	0.000	0.000	0.000	0.000	0.000	0.000
333	1194.938	0.003	2.390	0.003	2.390	0.003	2.390	0.003	2.390
334	1195.043	0.002	5.851	0.002	5.851	0.002	5.851	0.002	5.851
335	1195.500	0.003	3.872	0.003	3.872	0.003	3.872	0.003	3.872
336	1197.013	0.000	180.000	0.000	180.000	0.000	180.000	0.000	180.000
337	1197.140	0.000	0.000	0.000	0.000	0.000	0.000	0.000	0.000
338	1197.715	0.002	0.210	0.002	0.210	0.002	0.210	0.002	0.210
339	1198.755	0.000	0.000	0.000	0.000	0.000	0.000	0.000	0.000
340	1199.713	0.000	180.000	0.000	180.000	0.000	180.000	0.000	180.000
341	1288.508	0.000	0.000	0.000	0.000	0.000	0.000	0.000	0.000
342	1289.067	0.000	0.000	0.000	0.000	0.000	0.000	0.000	0.000
343	1289.526	0.000	0.000	0.000	0.000	0.000	0.000	0.000	0.000
344	1289.705	0.000	0.000	0.000	0.000	0.000	0.000	0.000	0.000
345	1293.320	0.001	171.648	0.001	171.648	0.001	171.648	0.001	171.648
346	1293.771	0.001	0.810	0.001	0.810	0.001	0.810	0.001	0.810
347	1295.727	0.000	0.000	0.000	0.000	0.000	0.000	0.000	0.000
348	1295.804	0.000	180.000	0.000	180.000	0.000	180.000	0.000	180.000
349	1296.030	0.001	5.392	0.001	5.392	0.001	5.392	0.001	5.392
350	1297.795	0.001	34.339	0.001	34.339	0.001	34.339	0.001	34.339
351	1298.329	0.002	0.219	0.002	0.219	0.002	0.219	0.002	0.219
352	1299.017	0.001	173.485	0.001	173.485	0.001	173.485	0.001	173.485
353	1301.023	0.000	180.000	0.000	180.000	0.000	180.000	0.000	180.000
354	1301.784	0.000	180.000	0.000	180.000	0.000	180.000	0.000	180.000
355	1302.249	0.000	0.000	0.000	0.000	0.000	0.000	0.000	0.000
356	1302.854	0.000	0.000	0.000	0.000	0.000	0.000	0.000	0.000
357	1303.221	0.000	0.000	0.000	0.000	0.000	0.000	0.000	0.000
358	1303.230	0.000	0.000	0.000	0.000	0.000	0.000	0.000	0.000
359	1303.737	0.000	180.000	0.000	180.000	0.000	180.000	0.000	180.000
360	1304.146	0.000	0.000	0.000	0.000	0.000	0.000	0.000	0.000
361	1304.240	0.001	14.061	0.001	14.061	0.001	14.061	0.001	14.061
362	1304.816	0.000	0.316	0.000	0.316	0.000	0.316	0.000	0.316

363	1304.883	0.003	1.172	0.003	1.172	0.003	1.172	0.003	1.172
364	1304.992	0.002	5.053	0.002	5.053	0.002	5.053	0.002	5.053
365	1310.694	0.000	143.533	0.000	143.533	0.000	143.533	0.000	143.533
366	1310.875	0.001	66.993	0.001	66.993	0.001	66.993	0.001	66.993
367	1311.058	0.000	28.045	0.000	28.045	0.000	28.045	0.000	28.045
368	1311.205	0.000	170.598	0.000	170.598	0.000	170.598	0.000	170.598
369	1318.160	0.000	180.000	0.000	180.000	0.000	180.000	0.000	180.000
370	1318.916	0.000	0.000	0.000	0.000	0.000	0.000	0.000	0.000
371	1320.089	0.003	3.862	0.003	3.862	0.003	3.862	0.003	3.862
372	1320.502	0.001	178.797	0.001	178.797	0.001	178.797	0.001	178.797
373	1363.146	0.000	0.000	0.000	0.000	0.000	0.000	0.000	0.000
374	1363.651	0.000	0.000	0.000	0.000	0.000	0.000	0.000	0.000
375	1364.100	0.000	130.950	0.000	130.950	0.000	130.950	0.000	130.950
376	1364.358	0.000	179.226	0.000	179.226	0.000	179.226	0.000	179.226
377	1364.566	0.000	0.000	0.000	0.000	0.000	0.000	0.000	0.000
378	1364.696	0.000	6.988	0.000	6.988	0.000	6.988	0.000	6.988
379	1364.742	0.000	45.525	0.000	45.525	0.000	45.525	0.000	45.525
380	1365.169	0.000	0.000	0.000	0.000	0.000	0.000	0.000	0.000
381	1367.804	0.000	180.000	0.000	180.000	0.000	180.000	0.000	180.000
382	1367.935	0.001	3.908	0.001	3.908	0.001	3.908	0.001	3.908
383	1368.591	0.000	180.000	0.000	180.000	0.000	180.000	0.000	180.000
384	1368.656	0.001	163.349	0.001	163.349	0.001	163.349	0.001	163.349
385	1389.799	0.000	180.000	0.000	180.000	0.000	180.000	0.000	180.000
386	1389.847	0.000	9.928	0.000	9.928	0.000	9.928	0.000	9.928
387	1390.132	0.000	40.472	0.000	40.472	0.000	40.472	0.000	40.472
388	1390.404	0.001	140.053	0.001	140.053	0.001	140.053	0.001	140.053
389	1390.472	0.000	0.000	0.000	0.000	0.000	0.000	0.000	0.000
390	1390.606	0.000	180.000	0.000	180.000	0.000	180.000	0.000	180.000
391	1390.830	0.000	0.000	0.000	0.000	0.000	0.000	0.000	0.000
392	1392.378	0.001	177.675	0.001	177.675	0.001	177.675	0.001	177.675
393	1393.439	0.003	0.482	0.003	0.482	0.003	0.482	0.003	0.482
394	1394.789	0.000	180.000	0.000	180.000	0.000	180.000	0.000	180.000
395	1394.822	0.000	180.000	0.000	180.000	0.000	180.000	0.000	180.000
396	1395.269	0.001	179.092	0.001	179.092	0.001	179.092	0.001	179.092
397	1398.609	0.001	3.893	0.001	3.893	0.001	3.893	0.001	3.893
398	1399.247	0.000	164.193	0.000	164.193	0.000	164.193	0.000	164.193
399	1400.241	0.001	22.141	0.001	22.141	0.001	22.141	0.001	22.141
400	1400.389	0.001	15.343	0.001	15.343	0.001	15.343	0.001	15.343
401	1403.727	0.000	180.000	0.000	180.000	0.000	180.000	0.000	180.000
402	1404.141	0.000	180.000	0.000	180.000	0.000	180.000	0.000	180.000
403	1413.813	0.001	108.343	0.001	108.343	0.001	108.343	0.001	108.343
404	1414.499	0.002	4.981	0.002	4.981	0.002	4.981	0.002	4.981
405	1414.893	0.000	0.000	0.000	0.000	0.000	0.000	0.000	0.000

406	1415.127	0.000	180.000	0.000	180.000	0.000	180.000	0.000	180.000
407	1416.156	0.000	180.000	0.000	180.000	0.000	180.000	0.000	180.000
408	1416.417	0.000	0.000	0.000	0.000	0.000	0.000	0.000	0.000
409	1420.016	0.000	0.000	0.000	0.000	0.000	0.000	0.000	0.000
410	1420.451	0.000	0.000	0.000	0.000	0.000	0.000	0.000	0.000
411	1421.237	0.000	0.000	0.000	0.000	0.000	0.000	0.000	0.000
412	1421.398	0.000	0.000	0.000	0.000	0.000	0.000	0.000	0.000
413	1427.411	0.000	180.000	0.000	180.000	0.000	180.000	0.000	180.000
414	1427.512	0.000	0.000	0.000	0.000	0.000	0.000	0.000	0.000
415	1430.527	0.000	0.000	0.000	0.000	0.000	0.000	0.000	0.000
416	1431.371	0.000	180.000	0.000	180.000	0.000	180.000	0.000	180.000
417	1432.011	0.002	10.116	0.002	10.116	0.002	10.116	0.002	10.116
418	1432.881	0.001	14.342	0.001	14.342	0.001	14.342	0.001	14.342
419	1433.406	0.001	4.856	0.001	4.856	0.001	4.856	0.001	4.856
420	1433.740	0.001	153.149	0.001	153.149	0.001	153.149	0.001	153.149
421	1433.951	0.001	179.095	0.001	179.095	0.001	179.095	0.001	179.095
422	1435.058	0.000	3.773	0.000	3.773	0.000	3.773	0.000	3.773
423	1436.082	0.000	178.914	0.000	178.914	0.000	178.914	0.000	178.914
424	1436.807	0.000	0.000	0.000	0.000	0.000	0.000	0.000	0.000
425	1437.154	0.000	0.000	0.000	0.000	0.000	0.000	0.000	0.000
426	1438.078	0.001	0.618	0.001	0.618	0.001	0.618	0.001	0.618
427	1438.351	0.000	0.000	0.000	0.000	0.000	0.000	0.000	0.000
428	1438.791	0.000	0.000	0.000	0.000	0.000	0.000	0.000	0.000
429	1440.200	0.000	180.000	0.000	180.000	0.000	180.000	0.000	180.000
430	1440.547	0.001	168.163	0.001	168.163	0.001	168.163	0.001	168.163
431	1441.277	0.000	0.000	0.000	0.000	0.000	0.000	0.000	0.000
432	1441.361	0.001	7.748	0.001	7.748	0.001	7.748	0.001	7.748
433	1448.539	0.000	0.000	0.000	0.000	0.000	0.000	0.000	0.000
434	1449.447	0.000	0.000	0.000	0.000	0.000	0.000	0.000	0.000
435	1449.817	0.000	180.000	0.000	180.000	0.000	180.000	0.000	180.000
436	1449.993	0.000	0.000	0.000	0.000	0.000	0.000	0.000	0.000
437	1450.103	0.000	103.254	0.000	103.254	0.000	103.254	0.000	103.254
438	1450.121	0.000	13.081	0.000	13.081	0.000	13.081	0.000	13.081
439	1450.425	0.000	172.684	0.000	172.684	0.000	172.684	0.000	172.684
440	1450.485	0.000	0.000	0.000	0.000	0.000	0.000	0.000	0.000
441	1450.942	0.000	180.000	0.000	180.000	0.000	180.000	0.000	180.000
442	1451.808	0.001	12.089	0.001	12.089	0.001	12.089	0.001	12.089
443	1452.618	0.000	180.000	0.000	180.000	0.000	180.000	0.000	180.000
444	1452.753	0.000	1.799	0.000	1.799	0.000	1.799	0.000	1.799
445	1453.453	0.000	180.000	0.000	180.000	0.000	180.000	0.000	180.000
446	1453.857	0.001	46.228	0.001	46.228	0.001	46.228	0.001	46.228
447	1454.865	0.000	136.914	0.000	136.914	0.000	136.914	0.000	136.914
448	1454.956	0.000	15.705	0.000	15.705	0.000	15.705	0.000	15.705

449	1471.075	0.000	0.000	0.000	0.000	0.000	0.000	0.000	0.000
450	1471.373	0.000	0.000	0.000	0.000	0.000	0.000	0.000	0.000
451	1472.727	0.000	0.000	0.000	0.000	0.000	0.000	0.000	0.000
452	1472.966	0.000	0.000	0.000	0.000	0.000	0.000	0.000	0.000
453	1542.806	0.002	14.399	0.002	14.399	0.002	14.399	0.002	14.399
454	1543.455	0.002	4.209	0.002	4.209	0.002	4.209	0.002	4.209
455	1549.062	0.002	144.994	0.002	144.994	0.002	144.994	0.002	144.994
456	1551.262	0.002	152.902	0.002	152.902	0.002	152.902	0.002	152.902
457	1557.274	0.000	0.000	0.000	0.000	0.000	0.000	0.000	0.000
458	1558.203	0.000	0.000	0.000	0.000	0.000	0.000	0.000	0.000
459	1558.797	0.000	0.000	0.000	0.000	0.000	0.000	0.000	0.000
460	1559.575	0.000	0.000	0.000	0.000	0.000	0.000	0.000	0.000
461	1563.562	0.000	161.096	0.000	161.096	0.000	161.096	0.000	161.096
462	1564.008	0.000	167.636	0.000	167.636	0.000	167.636	0.000	167.636
463	1564.563	0.001	10.450	0.001	10.450	0.001	10.450	0.001	10.450
464	1565.502	0.001	15.093	0.001	15.093	0.001	15.093	0.001	15.093
465	1581.340	0.001	16.280	0.001	16.280	0.001	16.280	0.001	16.280
466	1581.544	0.000	10.420	0.000	10.420	0.000	10.420	0.000	10.420
467	1583.079	0.000	5.696	0.000	5.696	0.000	5.696	0.000	5.696
468	1583.209	0.001	12.847	0.001	12.847	0.001	12.847	0.001	12.847
469	1592.788	0.000	34.285	0.000	34.285	0.000	34.285	0.000	34.285
470	1593.064	0.000	106.339	0.000	106.339	0.000	106.339	0.000	106.339
471	1595.127	0.000	36.311	0.000	36.311	0.000	36.311	0.000	36.311
472	1595.349	0.000	63.236	0.000	63.236	0.000	63.236	0.000	63.236
473	2598.617	0.000	164.264	0.000	164.264	0.000	164.264	0.000	164.264
474	2599.500	0.000	159.117	0.000	159.117	0.000	159.117	0.000	159.117
475	2612.913	0.000	62.926	0.000	62.926	0.000	62.926	0.000	62.926
476	2617.753	0.001	133.591	0.001	133.591	0.001	133.591	0.001	133.591
477	2709.429	0.001	7.109	0.001	7.109	0.001	7.109	0.001	7.109
478	2709.912	0.001	2.199	0.001	2.199	0.001	2.199	0.001	2.199
479	2720.113	0.001	13.033	0.001	13.033	0.001	13.033	0.001	13.033
480	2726.724	0.001	2.339	0.001	2.339	0.001	2.339	0.001	2.339
481	2802.910	0.000	175.411	0.000	175.411	0.000	175.411	0.000	175.411
482	2805.084	0.000	65.377	0.000	65.377	0.000	65.377	0.000	65.377
483	2806.606	0.001	173.285	0.001	173.285	0.001	173.285	0.001	173.285
484	2811.673	0.001	2.803	0.001	2.803	0.001	2.803	0.001	2.803
485	2965.136	0.000	0.000	0.000	0.000	0.000	0.000	0.000	0.000
486	2966.311	0.000	0.000	0.000	0.000	0.000	0.000	0.000	0.000
487	2969.274	0.001	138.912	0.001	138.912	0.001	138.912	0.001	138.912
488	2970.760	0.001	161.770	0.001	161.770	0.001	161.770	0.001	161.770
489	2970.862	0.000	0.000	0.000	0.000	0.000	0.000	0.000	0.000
490	2975.760	0.000	0.000	0.000	0.000	0.000	0.000	0.000	0.000
491	2975.941	0.001	169.642	0.001	169.642	0.001	169.642	0.001	169.642

492	2983.873	0.000	0.000	0.000	0.000	0.000	0.000	0.000	0.000
493	2983.936	0.000	0.000	0.000	0.000	0.000	0.000	0.000	0.000
494	2989.078	0.001	8.603	0.001	8.603	0.001	8.603	0.001	8.603
495	2991.983	0.000	0.000	0.000	0.000	0.000	0.000	0.000	0.000
496	2992.307	0.000	180.000	0.000	180.000	0.000	180.000	0.000	180.000
497	2992.462	0.000	180.000	0.000	180.000	0.000	180.000	0.000	180.000
498	2992.571	0.000	0.000	0.000	0.000	0.000	0.000	0.000	0.000
499	2994.291	0.000	7.207	0.000	7.207	0.000	7.207	0.000	7.207
500	2994.371	0.000	155.699	0.000	155.699	0.000	155.699	0.000	155.699
501	2994.511	0.000	125.647	0.000	125.647	0.000	125.647	0.000	125.647
502	2994.523	0.000	161.337	0.000	161.337	0.000	161.337	0.000	161.337
503	2996.440	0.000	180.000	0.000	180.000	0.000	180.000	0.000	180.000
504	2996.775	0.000	0.000	0.000	0.000	0.000	0.000	0.000	0.000
505	3002.060	0.000	0.000	0.000	0.000	0.000	0.000	0.000	0.000
506	3002.089	0.000	0.000	0.000	0.000	0.000	0.000	0.000	0.000
507	3002.267	0.000	0.000	0.000	0.000	0.000	0.000	0.000	0.000
508	3002.301	0.000	180.000	0.000	180.000	0.000	180.000	0.000	180.000
509	3002.790	0.000	165.192	0.000	165.192	0.000	165.192	0.000	165.192
510	3003.584	0.000	65.153	0.000	65.153	0.000	65.153	0.000	65.153
511	3003.628	0.000	16.824	0.000	16.824	0.000	16.824	0.000	16.824
512	3003.835	0.000	150.944	0.000	150.944	0.000	150.944	0.000	150.944
513	3008.030	0.000	28.752	0.000	28.752	0.000	28.752	0.000	28.752
514	3008.529	0.000	44.432	0.000	44.432	0.000	44.432	0.000	44.432
515	3008.591	0.000	168.892	0.000	168.892	0.000	168.892	0.000	168.892
516	3008.703	0.000	31.462	0.000	31.462	0.000	31.462	0.000	31.462
517	3010.608	0.000	180.000	0.000	180.000	0.000	180.000	0.000	180.000
518	3010.627	0.000	0.000	0.000	0.000	0.000	0.000	0.000	0.000
519	3013.541	0.000	67.578	0.000	67.578	0.000	67.578	0.000	67.578
520	3014.337	0.000	180.000	0.000	180.000	0.000	180.000	0.000	180.000
521	3014.388	0.000	158.621	0.000	158.621	0.000	158.621	0.000	158.621
522	3014.450	0.000	0.000	0.000	0.000	0.000	0.000	0.000	0.000
523	3016.845	0.000	180.000	0.000	180.000	0.000	180.000	0.000	180.000
524	3017.060	0.000	0.000	0.000	0.000	0.000	0.000	0.000	0.000
525	3017.361	0.000	51.403	0.000	51.403	0.000	51.403	0.000	51.403
526	3017.400	0.000	76.851	0.000	76.851	0.000	76.851	0.000	76.851
527	3019.740	0.000	0.000	0.000	0.000	0.000	0.000	0.000	0.000
528	3019.834	0.000	180.000	0.000	180.000	0.000	180.000	0.000	180.000
529	3024.393	0.000	126.814	0.000	126.814	0.000	126.814	0.000	126.814
530	3025.787	0.000	174.499	0.000	174.499	0.000	174.499	0.000	174.499
531	3030.712	0.000	19.554	0.000	19.554	0.000	19.554	0.000	19.554
532	3032.699	0.000	15.535	0.000	15.535	0.000	15.535	0.000	15.535
533	3055.567	0.000	0.000	0.000	0.000	0.000	0.000	0.000	0.000
534	3055.613	0.000	0.000	0.000	0.000	0.000	0.000	0.000	0.000

535	3055.747	0.000	0.000	0.000	0.000	0.000	0.000	0.000	0.000
536	3055.820	0.000	0.000	0.000	0.000	0.000	0.000	0.000	0.000
537	3056.409	0.000	175.498	0.000	175.498	0.000	175.498	0.000	175.498
538	3056.423	0.000	146.341	0.000	146.341	0.000	146.341	0.000	146.341
539	3056.751	0.000	110.215	0.000	110.215	0.000	110.215	0.000	110.215
540	3056.879	0.000	138.274	0.000	138.274	0.000	138.274	0.000	138.274
541	3070.489	0.000	0.000	0.000	0.000	0.000	0.000	0.000	0.000
542	3070.509	0.000	180.000	0.000	180.000	0.000	180.000	0.000	180.000
543	3071.128	0.000	180.000	0.000	180.000	0.000	180.000	0.000	180.000
544	3071.230	0.000	0.000	0.000	0.000	0.000	0.000	0.000	0.000
545	3071.323	0.000	24.884	0.000	24.884	0.000	24.884	0.000	24.884
546	3071.734	0.000	5.351	0.000	5.351	0.000	5.351	0.000	5.351
547	3072.085	0.000	25.171	0.000	25.171	0.000	25.171	0.000	25.171
548	3072.536	0.000	6.624	0.000	6.624	0.000	6.624	0.000	6.624
549	3079.872	0.000	180.000	0.000	180.000	0.000	180.000	0.000	180.000
550	3079.884	0.000	0.000	0.000	0.000	0.000	0.000	0.000	0.000
551	3080.282	0.000	35.350	0.000	35.350	0.000	35.350	0.000	35.350
552	3080.377	0.000	1.951	0.000	1.951	0.000	1.951	0.000	1.951
553	3081.193	0.000	0.000	0.000	0.000	0.000	0.000	0.000	0.000
554	3081.240	0.000	180.000	0.000	180.000	0.000	180.000	0.000	180.000
555	3082.403	0.000	47.682	0.000	47.682	0.000	47.682	0.000	47.682
556	3082.512	0.000	8.428	0.000	8.428	0.000	8.428	0.000	8.428
557	3082.767	0.000	134.353	0.000	134.353	0.000	134.353	0.000	134.353
558	3082.827	0.000	40.736	0.000	40.736	0.000	40.736	0.000	40.736
559	3082.874	0.000	0.000	0.000	0.000	0.000	0.000	0.000	0.000
560	3082.897	0.000	0.000	0.000	0.000	0.000	0.000	0.000	0.000
561	3083.008	0.000	17.197	0.000	17.197	0.000	17.197	0.000	17.197
562	3083.049	0.000	180.000	0.000	180.000	0.000	180.000	0.000	180.000
563	3083.064	0.000	180.000	0.000	180.000	0.000	180.000	0.000	180.000
564	3083.134	0.000	29.177	0.000	29.177	0.000	29.177	0.000	29.177
565	3161.554	0.000	137.385	0.000	137.385	0.000	137.385	0.000	137.385
566	3162.106	0.000	159.515	0.000	159.515	0.000	159.515	0.000	159.515
567	3163.278	0.000	129.547	0.000	129.547	0.000	129.547	0.000	129.547
568	3166.046	0.000	168.822	0.000	168.822	0.000	168.822	0.000	168.822
569	3351.805	0.001	15.769	0.001	15.769	0.001	15.769	0.001	15.769
570	3352.066	0.001	20.197	0.001	20.197	0.001	20.197	0.001	20.197
571	3359.524	0.001	161.011	0.001	161.011	0.001	161.011	0.001	161.011
572	3361.041	0.001	157.848	0.001	157.848	0.001	157.848	0.001	157.848
573	3365.294	0.000	0.000	0.000	0.000	0.000	0.000	0.000	0.000
574	3366.206	0.000	0.000	0.000	0.000	0.000	0.000	0.000	0.000
575	3366.284	0.000	0.000	0.000	0.000	0.000	0.000	0.000	0.000
576	3368.074	0.001	0.000	0.001	0.000	0.001	0.000	0.001	0.000

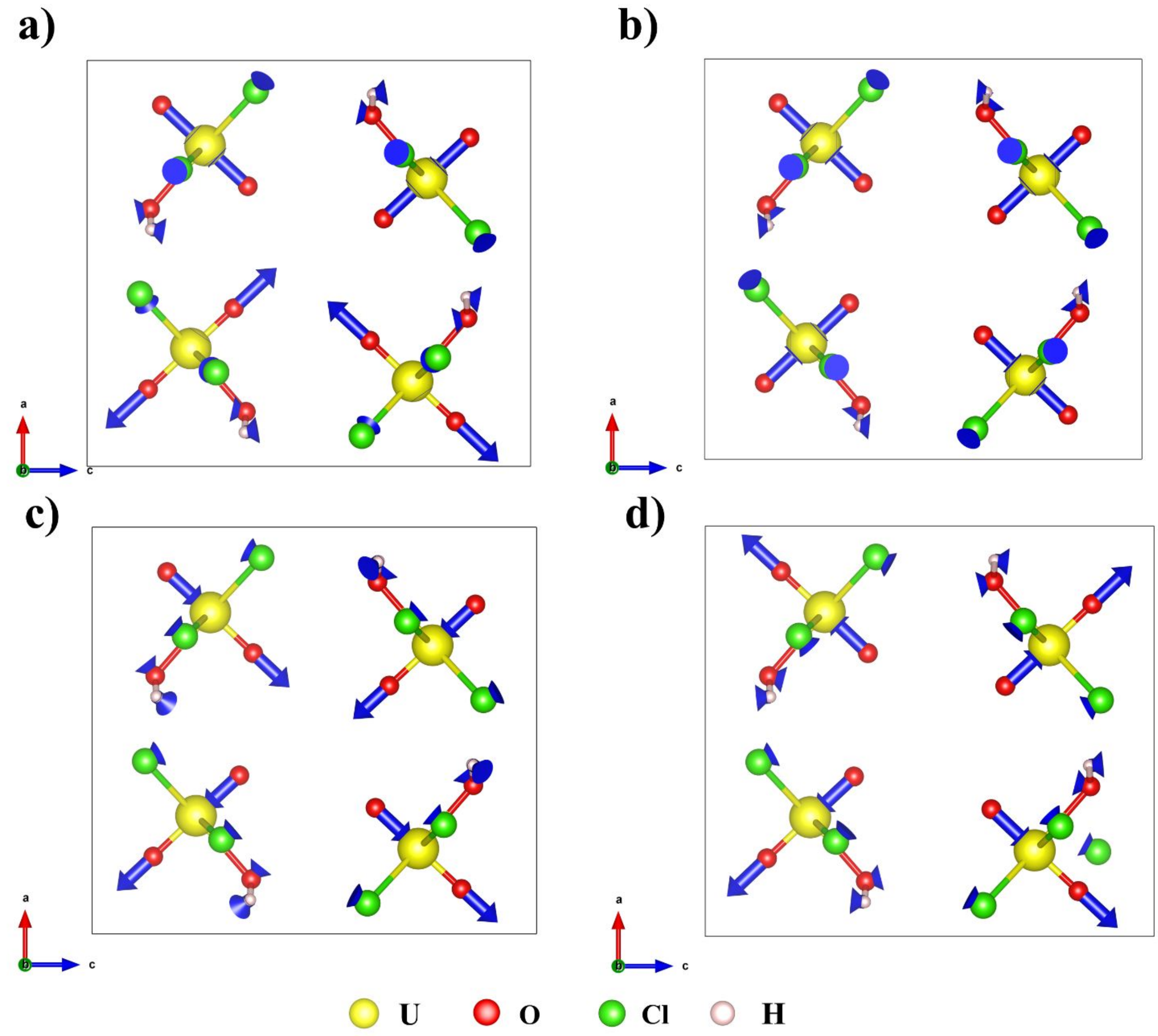

Figure S5: Phonon eigenvectors of normal modes centered at 825 cm^{-1}, 832 cm^{-1}, 990 cm^{-1}, and 1005 cm^{-1} are shown in figure a, b, c, and d respectively. The piperazinium cation and uncoordinated chlorides contribute slightly to the normal mode, thus they have not displayed her to make the image appear clear.

7. Thermochemistry

Table S7: Solvation enthalpies of $(C_4H_{12}N_2)_2[UO_2Cl_4(H_2O)]Cl_{2(s)}$ in 2N HCl at 25.0 ± 0.1 °C and ambient pressure.

**Experiment number**	**Mass of the drop (mg)**	**ΔH_{sol} (kJ/mol)**
1	11.1	-30.72
2	19.1	-30.18
3	19.8	-30.31

8. References

(1) Manz, T. A.; Limas, N. G. Introducing DDEC6 atomic population analysis: part 1. Charge partitioning theory and methodology. *RSC Advances* **2016**, *6* (53), 47771-47801, 10.1039/C6RA04656H. DOI: 10.1039/C6RA04656H.
(2) Limas, N. G.; Manz, T. A. Introducing DDEC6 atomic population analysis: part 2. Computed results for a wide range of periodic and nonperiodic materials. *RSC Advances* **2016**, *6* (51), 45727-45747, 10.1039/C6RA05507A. DOI: 10.1039/C6RA05507A.
(3) Manz, T. A. Introducing DDEC6 atomic population analysis: part 3. Comprehensive method to compute bond orders. *RSC Advances* **2017**, *7* (72), 45552-45581, 10.1039/C7RA07400J. DOI: 10.1039/C7RA07400J.
(4) Limas, N. G.; Manz, T. A. Introducing DDEC6 atomic population analysis: part 4. Efficient parallel computation of net atomic charges, atomic spin moments, bond orders, and more. *RSC Advances* **2018**, *8* (5), 2678-2707, 10.1039/C7RA11829E. DOI: 10.1039/C7RA11829E.
(5) Spano, T. L.; Shields, A. E.; Barth, B. S.; Gruidl, J. D.; Niedziela, J. L.; Kapsimalis, R. J.; Miskowiec, A. Computationally Guided Investigation of the Optical Spectra of Pure β-UO3. *Inorganic Chemistry* **2020**, *59* (16), 11481-11492. DOI: 10.1021/acs.inorgchem.0c01279.
(6) Augustine, L. J.; Rajapaksha, H.; Pyrch, M. M. F.; Kasperski, M.; Forbes, T. Z.; Mason, S. E. Periodic Density Functional Theory Calculations of Uranyl Tetrachloride Compounds Engaged in Uranyl–Cation and Uranyl–Hydrogen Interactions: Electronic Structure, Vibrational, and Thermodynamic Analyses. *Inorganic Chemistry* **2023**, *62*, 372-380. DOI: 10.1021/acs.inorgchem.2c03476.
